# Evaluating the Diagnostic Performance of Long-Read Metagenomic Sequencing Compared to Culture and Antimicrobial Susceptibility Testing for Detection of Bovine Respiratory Bacteria and Indicators of Antimicrobial Resistance

**DOI:** 10.3390/antibiotics14111114

**Published:** 2025-11-05

**Authors:** Jennifer N. Abi Younes, Lianne McLeod, Simon J. G. Otto, Zhijian Chai, Stacey Lacoste, E. Luke McCarthy, Matthew G. Links, Emily K. Herman, Paul Stothard, Sheryl P. Gow, John R. Campbell, Cheryl L. Waldner

**Affiliations:** 1 Department of Large Animal Clinical Sciences, Western College of Veterinary Medicine, University of Saskatchewan, Saskatoon, SK S7N 5B4, Canada; 2 HEAT-AMR (Human-Environment-Animal Transdisciplinary AMR) Research Group, School of Public Health, University of Alberta, Edmonton, AB T6G 2J7, Canada; 3 Centre for Healthy Communities, School of Public Health, University of Alberta, Edmonton, AB T6G 1C9, Canada; 4 Department of Animal and Poultry Science, College of Agriculture and Bioresources, University of Saskatchewan, Saskatoon, SK S7N 5A8, Canada; 5 Department of Biochemistry and Medical Genetics, Max Rady College of Medicine, University of Manitoba, Winnipeg, MB R3E 0J9, Canada; 6 Department of Agricultural, Food, and Nutritional Science, Faculty of Agricultural, Life, and Environmental Sciences, University of Alberta, Edmonton, AB T6G 2P5, Canada; 7 Canadian Integrated Program for Antimicrobial Resistance Surveillance, Public Health Agency of Canada, Saskatoon, SK S7N 5B4, Canada

**Keywords:** bovine respiratory disease, bacteria, antimicrobial resistance, antimicrobial resistance genes, long-read metagenomic sequencing, feedlot calves

## Abstract

**Background/Objectives:** Long-read metagenomic sequencing can detect bacteria and antimicrobial resistance genes (ARGs) from bovine respiratory samples, providing an alternative to culture and antimicrobial susceptibility testing (C/S). This study applied Bayesian latent class models (BLCMs) to estimate the sensitivity (Se) and specificity (Sp) of long-read metagenomic sequencing compared to C/S for detecting *Mannheimia haemolytica*, *Pasteurella multocida*, and *Histophilus somni*, as well as associated macrolide and tetracycline resistance potential. **Methods:** Deep nasopharyngeal swabs were collected from fall-placed feedlot calves at arrival, 13, and 36 days on feed across two years and two metaphylaxis protocols. Samples underwent C/S and long-read metagenomic sequencing. BLCMs were used to estimate Se and Sp for the detection of bacteria and potential for antimicrobial resistance (AMR). **Results:** Se and Sp for detecting respiratory bacteria by metagenomics were not significantly different than culture, with four exceptions. For the 2020 samples, Se for *M. haemolytica* was lower than culture, and Sp for *H. somni* was lower, while in both 2020 and 2021 samples, Se for *P. multocida* was higher for metagenomics than culture. The estimated Se and Sp of metagenomics for the detection of *msrE-mphE*, *EstT*, and *tet(H)* within bacterial reads were either not significantly different or were lower than AST, with Sp > 95% with one exception. **Conclusions:** This study provided BLCM-based estimates of clinical Se and Sp of metagenomics and C/S without assuming a gold standard in a large pen research setting. These findings demonstrate the potential of long-read metagenomics to support bovine respiratory disease diagnostics, AMR surveillance, and antimicrobial stewardship in feedlot cattle.

## 1. Introduction

Diagnostic tools are the cornerstone of effective disease management and treatment. However, for bacterial diseases, laboratory diagnostics for detecting antimicrobial resistance (AMR) in key bacterial species is often hindered by slow turnaround times, reliance on the recovery of viable organisms, and extensive, multi-step methods for identifying AMR [1,2,3]. These inefficiencies limit the use of testing in an era of growing emphasis on laboratory support for prudent antimicrobial use [4,5,6]. As a result, new laboratory tools are being explored, including long-read metagenomics, due to its ability to simultaneously detect bacteria of interest and associated antimicrobial resistance genes (ARGs) on a single metagenomic sequencing read [7].

In North American feedlot cattle, bovine respiratory disease (BRD) remains the leading indication for parenteral antimicrobial use (AMU) [8,9]. While antimicrobials are critical for control and treatment of this disease, a global focus on reducing AMU in food-producing animals has emerged in response to rising antimicrobial resistance (AMR) in both humans and animals [4]. As such, BRD is a focus of antimicrobial stewardship initiatives and research, with an emphasis on the use of diagnostic testing to guide antimicrobial decision-making [10,11].

Traditionally, culture and antimicrobial susceptibility testing (AST) have been widely regarded as the gold standard for detection of bacteria and AMR [12] and used as references for assessing other laboratory tools [13,14,15]. However, culture-based methods are inherently dependent on bacterial growth necessary to first identify individual colonies, followed by a second step to evaluate antimicrobial susceptibility [3]. The interpretation of AST results is also limited, as evidence-based breakpoints to classify resistance status have not been established for all antimicrobial bacterial combinations of interest, particularly for veterinary pathogens [16]; however, these breakpoints are reviewed and updated as new information becomes available.

Long-read metagenomic sequencing has the potential to identify ARGs within speciated bacterial reads [3,7], directly linking resistance mechanisms to BRD bacteria of interest in one step. Conventional molecular methods, such as polymerase chain reaction (PCR), are not suitable for linking ARGs to bacterial species [17]. While sequencing methods, such as short-read metagenomic sequencing, can putatively link ARGs to the bacteria of interest, this typically requires large amounts of short-read data and computational resources to assemble the reads and then link ARGs to taxa in the assembled sequence. In the context of BRD, many of the primary bacterial pathogens of interest, including *Mannheimia haemolytica*, *Pasteurella multocida*, and *Histophilus somni*, are also commensals of the upper respiratory tract. Species-level links of ARGs to the pathogens of interest are also highly relevant due to the potential for horizontal gene transfer among these organisms [18,19,20,21]. Therefore, long-read metagenomic sequencing facilitates direct and confident identification of AMR potential in clinically relevant bacteria, supporting clinical decisions, surveillance, and antimicrobial stewardship objectives.

While culture and AST have been used as a reference, they do not represent a true gold standard for diagnosis [13,14,15]. The absence of a true gold standard for diagnostic testing creates challenges in objectively evaluating newer technologies like metagenomic sequencing [22]. Bayesian latent class models (BLCMs) can be used to estimate diagnostic sensitivity (Se) and specificity (Sp) from samples collected under field conditions, as well as true prevalence, without requiring a ‘perfect’ test reference [23,24]. These models leverage cross-classification of outcomes from multiple laboratory tests using data from multiple populations and prior knowledge, when available, to improve model identifiability [25,26]. Notably, the World Organization for Animal Health (OIE) emphasizes the value of BLCMs for evaluating the performance of diagnostic tests for infectious diseases and considers them a valid method for test validation [27].

The objective of this study was to use BLCMs to estimate the diagnostic performance of long-read metagenomic sequencing, compared to culture and AST, for detecting bacteria and AMR associated with bacterial BRD organisms in respiratory samples from feedlot cattle. Measures of resistance were evaluated for antimicrobials commonly used to manage BRD in Canadian feedlot cattle [9] and where phenotypic resistance had been previously reported [28,29]. Changes in the prevalence based on metagenomic sequencing data were examined across time and metaphylaxis types to evaluate important assumptions of the BLCMs and compare the observed differences to those previously reported from concurrent phenotype data [29]. The findings from this study can be used to assess the potential application of long-read metagenomic sequencing for detection of BRD-associated bacteria and AMR to advance BRD diagnostics and support AMR detection strategies.

## 2. Results

A total of 1988 samples with long-read metagenomic sequencing were included in this study, consisting of 909 samples from 2020 and 1079 in 2021, with matched culture data. Of these, complete AST data were available for all samples from 2020 and all but 3 from 2021. Samples included 840 (838 with complete AST data) collected at 1 DOF and prior to metaphylaxis administration, 819 at 13 DOF, and 329 (328 with complete AST data) at 36 DOF.

### 2.1. Detection of Target Bacteria via Long-Read Metagenomic Sequencing

Sequencing performance metrics across all samples were summarized in Table 1 and included total base pairs, sample median read length, theoretical coverage, and number of reads, as well as the percentage of samples with at least one read detected for each of *M. haemolytica*, *P. multocida*, and *H. somni*.

Coverage was highest for *M. haemolytica*, followed by *P. multocida*, then *H. somni* in both years (Table 1). At least one read of *M. haemolytica* was detected in all samples and *P. multocida* in almost all samples. Fewer *H. somni* reads were detected and in a smaller proportion of samples. Median sample read length was higher for the 2021 samples, which were processed fresh; however, the total number of reads of target organisms detected from the 2021 samples was less than 2020. As a result, differences were minimal in theoretical coverage between the 2020 and 2021 metagenomic sequencing data.

### 2.2. Detection of Antimicrobial Resistance Genes in Bacterial Reads

Across all samples from both years, 127 unique ARGs were identified within *M. haemolytica*, *P. multocida*, and *H. somni* reads from 1985 sequenced samples with phenotypic AMR data (Table S2). Any ARGs found in >100 samples across both years are listed in Table 2; all other ARGs were identified in fewer than 35 samples.

The most prevalent and potentially clinically relevant ARGs for consideration in BLCMs were *tet(H)*, *mphE*, *msrE*, and *EstT* (Table 2).

Detection of reads containing *msrE-mphE*, *EstT*, and *tet(H)* varied among the target bacterial species, time points, sampling years, and metaphylaxis groups (Table 3).

While *sul2* genes were common in these samples, particularly in 2020, dihydrofolate reductase (DHFR) genes resulting in resistance to trimethoprim were identified in less than 5 samples (Table S2).

Additional ARGs of potential clinical interest, including *CRP* (efflux pump), *H-NS* (efflux pump), and *floR* (phenicols), were detected in 1.5% of samples or less (Table S2). Other genes associated with tetracycline or macrolide resistance were also very rare, with *Erm(42)* and *tet(38)* observed in fewer than 1% of samples.

### 2.3. Phenotypic Non-Susceptibility

A previous summary of AST results in this population, where intermediate MICs were classified as susceptible, can be found in Abi Younes et al. [29]. For the present study using BLCM to cross-classify ARGs with AST results, the most frequent and corresponding phenotypic non-susceptibilities, supported by CLSI breakpoints, were for macrolides (all tested macrolides, 15-membered ring (gamithromycin and tulathromycin), and 16-membered ring (tildipirosin and tilmicosin)), and tetracycline (Table 4).

### 2.4. Bayesian Latent Class Models for Detection of BRD Bacteria

For 2020, the threshold determined by ROC analysis met the criteria of a BLCM-estimated diagnostic Sp ≥ 0.90 for *M. haemolytica* (5.1× coverage) and *P. multocida* (1.2× coverage), while the ROC-determined threshold was increased five-fold to meet the minimum Sp of 0.90 in the BLCM for *H. somni* (final cutoff of 0.09× coverage) (Table 5). In 2021, the threshold determined by ROC analysis met the BLCM-estimated diagnostic Sp ≥ 0.90 target for *M. haemolytica* (1.7× coverage), *P. multocida* (0.26× coverage), and *H. somni* (0.05× coverage).

The estimated diagnostic Se of culture for the detection of *M. haemolytica* based on BLCM was higher than for long-read sequencing for the analysis of the 2020 samples, while the sensitivities of culture and metagenomic sequencing were comparable for the 2021 data (Table 6). The estimated Se of long-read sequencing was higher for the 2021 metagenomic data than for the 2020 metagenomic analysis. Test specificities for the detection of *M. haemolytica* were comparable between tests and years with overlapping 95% CrIs (Table 6).

Conversely, for the detection of *P. multocida*, the estimated Se of long-read metagenomics was higher than that of culture in both the 2020 and 2021 samples (Table 6), with no significant difference between years for Se based on metagenomic analysis. The specificities for detection of *P. multocida* were similar between the tests as well as between years (Table 6).

For the detection of *H. somni*, the Se of long-read metagenomic sequencing [0.52 (95% CrI: 0.36–0.67)] tended to be slightly lower than that of culture [0.84 (95% CrI: 0.65–0.999)] in the 2020 samples but was more comparable in the 2021 samples (Table 6). However, like the results for *M. haemolytica*, the Se of metagenomic sequencing for the detection of *H. somni* was higher in the 2021 samples than in the 2020 samples. Similarly, the Sp of metagenomic sequencing for detection of *H. somni* was slightly lower in 2020 [0.90 (95% CrI: 0.88–0.92)] than the Sp of culture in 2020 [0.97 (95% CrI: 0.95–0.99)] as well as the Sp for metagenomic sequencing in 2021. The Sp of metagenomic sequencing and culture were comparable in the 2021 samples (Table 6).

The percentage of samples with theoretical coverage above the cut-off developed by ROC analysis combined with BLCM were summarized by year, metaphylaxis, and time of sampling for each of the bacterial species of interest (Table 5).

### 2.5. Bayesian Latent Class Models for Detection of Antimicrobial Non-Susceptibility in BRD Bacteria

In the 2020 samples, the diagnostic Se of AST derived from the BLCM for detecting non-susceptibility to *any* macrolide in *M. haemolytica, P. multocida* and/or *H. somni* [0.86 (95% CrI: 0.80–0.92)] was higher than metagenomic sequencing-based detection of *msrE-mphE* genes in any of these bacteria [0.61 (95% CrI: 0.55–0.68)] (Table 7).

Similarly, in the model comparing detection of AST-defined non-susceptibility to 15-membered macrolides (gamithromycin or tulathromycin) to *msrE-mphE*, AST Se was higher than the Se of metagenomic sequencing (Table 7). Estimates of Se from the BLCM with AST to 16-membered ring macrolides (tildipirosin or tilmicosin) and metagenomic sequencing detection of *msrE-mphE* were comparable and had overlapping CrIs. However, the Se of AST was lower for 16- vs. 15-membered ring macrolides. The Se and Sp for metagenomic identification of *msrE-mphE* did not differ depending on whether the second tests in the BLCM AST for were 15- or AST for 16-membered ring macrolides. Results were not available for the *msrE-mphE* models in 2021 due to a low number of samples (3-*M. haemolytica*, 1-*H. somni*) with any *msrE* or *mphE* reads.

For the 2020 analysis, the diagnostic Se of metagenomic sequencing for detection of *EstT* based on BLCM was significantly less than for AST for detection of non-susceptibility to any macrolide, 15-membered ring or 16-membered ring macrolides (Table 7). The individual test Se was similar for metagenomic detection of *EstT* and AST, when comparing any macrolides, 15-membered ring, and 16-membered ring macrolides (all CrIs overlapped). However, in the 2021 samples, the CrI for Se of metagenomic detection of *EstT* overlapped with AST for non-susceptibility for any macrolides and 16-membered ring macrolide models, but metagenomic detection of *EstT* had higher Se than AST for the 15-membered ring macrolides (Table 7). The Sp for all comparisons of AST and *EstT* detection were high, and credible intervals overlapped for all years and comparisons (Table 7).

For the detection of any macrolide non-susceptibility using combined metagenomics detection of *msrE-mphE* and/or *EstT*, metagenomic Se improved relative to the individual gene model for *EstT* in the 2020 samples but not in the 2021 samples or for *msrE-mphE* in the 2020 samples (Table 7). Credible intervals for metagenomic sequencing and AST overlapped in the 2021 analyses. Specificities for both methods remained high between years.

For the detection of tetracycline non-susceptibility, metagenomic sequencing detection of *tet(H)* and AST demonstrated similar Se with overlapping CrIs in both years (Table 7). In the 2020 samples, the Sp of AST was higher than that of metagenomic sequencing detection of *tet(H)* (non-overlapping CrIs), while specificities were comparable in the 2021 samples (Table 7).

**Figure 1:**
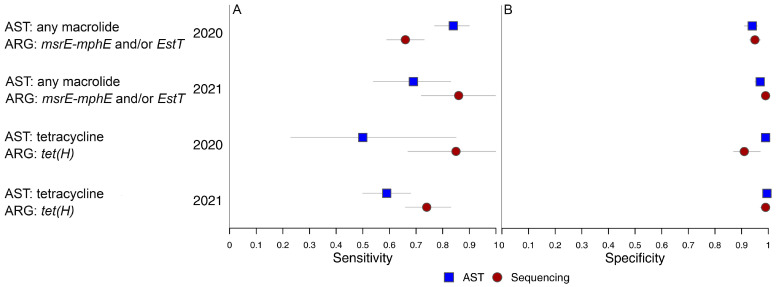
Plot of Bayesian latent class model estimates of sensitivity (**A**) and specificity (**B**) of antimicrobial susceptibility testing (AST) and long-read metagenomic sequencing for models comparing macrolide non-susceptibility classified by AST and detection of *msrE-mphE* and/or *EstT* by metagenomics, and tetracycline non-susceptibility classified by AST and the detection of *tet(H)* by metagenomics.

Comparisons among the most clinically relevant estimates from the BLCMs were summarized in Figure 1.

### 2.6. Sensitivity Analyses Examining the Assumptions of the BLCM

When assessing the sensitivity of the BLCMs for bacterial detection to the assumption of conditional independence between tests, the median estimates and the CrIs between the models with (Table S3) and without (Table 6) covariance terms remained largely similar. There were some shifts in the median estimates for Se and Sp for each test, but with considerable overlap in their credible intervals. Additionally, the 95% credible intervals for the covariance terms included zero, suggesting that the BLCMs were not significantly influenced by potential dependence between culture and metagenomics introduced by the enrichment step prior to metagenomic sequencing. Models without covariance terms were chosen for the final most parsimonious estimates of test performance.

Comparison to models with intermediate AST results classified as susceptible rather than resistant revealed only a few differences that varied across resistance types. Differences were minimal in the tetracycline-*tet(H)* model (Table S4) and most macrolide models. However, in the tildipirosin/tilmicosin-*EstT* model for 2020, the Se for metagenomic sequencing of *EstT* was significantly higher for the model classifying intermediate with susceptible (Se 0.46 (95% CrI 0.24–0.70)) compared to the model classifying intermediate with resistant (Se 0.15 (95% CrI 0.10–0.20)).

Excluding the on-arrival population had little effect on the estimates of test Se and Sp in models for the detection of bacteria (Table S5) as well as those for the detection of AMR and ARG (Table S6).

In a final sensitivity analysis, Se and Sp estimates from models for resistance for individual macrolides determined by AST for *M. haemolytica* were compared to the detection of specific ARGs (tulathromycin: *msrE-mphE*, tilmicosin: *EstT*) identified on *M. haemolytica* reads for 2020 data (Table S7). Estimates were very similar to those from models comparing grouped phenotypic AST results and detection of *msrE-mphE* and the model comparing tilmicosin or tildipirosin AST to *EstT* in any of *M. haemolytica, P. multocida* or *H. somni* (Table 7).

### 2.7. Impacts of Se and Sp on Positive and Negative Predictive Value Based on Long-Read Metagenomic Sequencing

Based on the results from the BLCMs for 2020 and 2021, estimates for PPV exceeded 75% across both years when prior probability of detection exceeded 0.25 for *M. haemolytica*, 0.10 for *P. multocida*, and 0.40 for *H. somni* (Table S8a–c). Similarly, estimates for NPV exceeded 75% across both years when prior probability of detection was less than 0.50 for *M. haemolytica*, 0.75 for *P. multocida*, and 0.30 for *H. somni* (Table S8a–c).

Available estimates for PPV for detection of non-susceptibility exceeded 75% when prior probability of detection exceeded 0.15 for *msrE-mphE* for any macrolide, 0.40 for *EstT* for any macrolide, and 0.25 for *tet(H)* for tetracycline (Table S9a–c). Similarly, available estimates for NPV for detection of lack of susceptibility exceeded 75% when prior probability of detection was less than 0.40 for *msrE-mphE* for any macrolide, 0.25 for *EstT* for any macrolide, and 0.50 for *tet(H)* for tetracycline (Table S9a–c).

### 2.8. Differences in Detection of BRD-Associated Bacteria over Time Based on Long-Read Metagenomic Sequencing

Among calves receiving tulathromycin metaphylaxis in 2020, the likelihood of detecting *M. haemolytica* with long-read metagenomics were around 5 times greater at 36 DOF compared to both 1 DOF and 13 DOF (Table 8). There was no significant difference between 13 DOF and 1 DOF. In 2021 tulathromycin calves, *M. haemolytica* detection decreased from 1 DOF to 13 DOF, then increased significantly from 13 DOF to 36 DOF (Table 8). Among calves receiving oxytetracycline in 2021, *M. haemolytica* detection increased slightly from 1 DOF to 13 DOF but did not change significantly thereafter (Table 8).

For *P. multocida*, metagenomic-based detection declined sharply between 1 DOF and 13 DOF in both years for the pens of calves receiving tulathromycin metaphylaxis, but not in 2021 for the calves receiving oxytetracycline (Table 8). However, detection of *P. multocida* then increased from 13 to 36 DOF in both years for all metaphylaxis groups. For tulathromycin-treated groups in 2020 and 2021, the detection of *P. multocida* remained less frequent at 36 DOF compared to 1 DOF. In calves receiving oxytetracycline metaphylaxis in 2021, *P. multocida* detection increased from 1 DOF to 36 DOF.

Metagenomic sequencing-based detection of *H. somni* increased substantially over time in all groups, between 1 DOF and 36 DOF and 13 DOF and 36 DOF (Table 8). *H. somni* was also more frequently detected at 13 DOF than 1 DOF in 2021 in the calves receiving oxytetracycline metaphylaxis, but not for the calves receiving tulathromycin in either 2020 or 2021.

### 2.9. Differences in Detection of ARGs in BRD-Associated Bacteria over Time

In 2020, detection of *mphE-msrE* increased significantly after arrival. The likelihood of detecting *mphE-msrE* was 20 times higher at 13 DOF compared to 1 DOF, and 42 times higher at 36 DOF compared to 1 DOF (Table 9). A significant increase was also observed between 13 DOF and 36 DOF. In contrast, *mphE-msrE* detection in 2021 was very limited (Table 2). Among tulathromycin-treated calves, there were no significant differences over time (Table 9). No *mphE-msrE* positive samples were detected in oxytetracycline-treated calves in 2021.

While the detection of *mphE-msrE* varied substantially from 2020 to 2021, there was no measurable difference in *EstT* detected between 2020 and 2021. For *EstT*, detection increased between 1 DOF and 13 DOF and 1 DOF and 36 DOF in calves receiving tulathromycin metaphylaxis in both 2020 and 2021 (Table 9). A modest but significant increase between 13 DOF and 36 DOF was also observed in 2021. Among the calves receiving oxytetracycline metaphylaxis, there were no significant increases in detection of *EstT* over time.

For *tet(H*), detection increased significantly from 1 DOF to 36 DOF in all groups in both 2020 and 2021 (Table 9). There were also increases in the detection of *tet(H)* from 13 DOF to 36 DOF in calves receiving tulathromycin in both 2020 and 2021. Finally, in 2021 *tet(H)* increased significantly between 1 DOF and 13 DOF in both metaphylaxis groups.

## 3. Discussion

This study applied BLCMs to evaluate the diagnostic performance of long-read metagenomic sequencing and culture for detecting *M. haemolytica*, *P. multocida*, *H. somni*, as well as metagenomics and AST for associated ARGs and AMR recovered from DNPS samples of fall-placed calves in a research feedlot. The resulting estimates of Se and Sp provide insight on their potential to evaluate AMR in bacteria most associated with BRD early in the feeding period. The scale of this study relative to previous bacterial metagenomic studies of BRD in feedlot cattle [7,15,30,31,32], the target population of recently weaned, highly mixed auction sourced calves, repeated testing early in the feeding period, and more typical pen size of the research feedlot contributes foundational data for understanding the role of metagenomic sequencing in clinical decision-making, surveillance, and antimicrobial stewardship initiatives.

Application of BLCMs revealed that in 2021 samples processed without freezing, Se estimates for long-read metagenomic sequencing and culture were similar for *M. haemolytica*, *P. multocida*, and *H. somni*. However, in 2020, when samples were frozen prior to processing, the Se of metagenomic sequencing for *M. haemolytica* was lower than that for culture but higher for *P. multocida*. For *H. somni*, the difference between sequencing and culture was not statistically significant. The Se of metagenomic detection of both *M. haemolytica* and *H. somni* detection was lower for the frozen 2020 samples as compared to the fresh 2021 samples, but there was no difference for *P. multocida*. While we cannot definitively attribute the differences to freezing the 2020 samples, freezing has been demonstrated to limit recovery in culture-based studies [33,34,35,36,37,38,39]. This was relevant to metagenomic sequencing in the current protocol because of the importance of the bacterial enrichment step in recovery of sufficient amounts of target organism sequence data to identify ARGs attributed to target bacterial species [7]. The performance of culture did not vary between years, as samples from both 2020 and 2021 were transported in temperature-controlled coolers and cultured on the same day as collection [33,40]. The proximity of the collection site to the lab likely positively contributed to the estimated Se. By contrast, typical field-collected diagnostic samples may experience transport delays and temperature fluctuations before reaching the lab, reducing viability.

The estimated Se results varied more substantially across AMR and ARG targets than for detection of organisms. Metagenomic sequencing generally demonstrated lower Se than AST for detecting macrolide non-susceptibility in the 2020 samples. This was especially pronounced for *EstT* detection in 2020, where the Se of sequencing was very low for detecting non-susceptibility to macrolides. However, the Se of metagenomic sequencing for the detection of *EstT* increased substantially in 2021. The Se of metagenomic detection of *mphE-msrE* and AST for detection of macrolide non-susceptibility were moderately higher, but estimates were only available for 2020. The Se estimates for *mphE-msrE* detection were slightly lower than AST for gamithromycin and tulathromycin non-susceptibility, but both Se estimates were similar in a model for tildipirosin and tilmicosin. In 2021, BLCMs considering *mphE-msrE* and macrolide non-susceptibility could not be evaluated due to the low frequency of *mphE-msrE* detection and overall lower macrolide resistance burden. For tetracycline non-susceptibility, the Se of metagenomic sequencing detection of *tet(H)* was not significantly different than AST. The detection of *tet(H)* on reads of *M. haemolytica*, *P. multocida* or *H. somni* had moderately good Se with high to very high Sp for the detection of tetracycline non-susceptibility across both study years.

The estimates of Se for *EstT* for the detection of 16-membered ring macrolides appeared to have been limited by the choice to classify intermediate phenotypes with resistant as non-susceptible in this analysis. The resulting Se of *EstT* for detection of 16-membered ring macrolide resistance was significantly higher in the 2020 samples when intermediate MIC values were classified as susceptible in a supplemental analysis. This was the only case where classifying intermediate MIC results as susceptible resulted in significantly different estimates for metagenomic sequencing.

While Se and Sp provide indicators of intrinsic performance of the test itself, clinicians in practice are ultimately concerned with the probability that a positive or negative test result correctly reflects the true presence or absence of bacteria and potential for antimicrobial resistance. These probabilities are described by PPV and NPV and are influenced by Se and Sp as well as the underlying prevalence (pretest probability) [41]. In this study, the PPV of all assays was greater than 75% when pretest probability exceeded 0.40, and the NPV was greater than 75% when pretest probability was less than 0.25. Therefore, positive metagenomic results will be most informative for confirming bacterial resistance in moderate to high-prevalence scenarios and for ruling out resistance in lower-prevalence scenarios.

While this study used BLCMs to compare the diagnostic performance of AST and long-read metagenomic sequencing, others have made comparisons between AST and other laboratory tests for AMR in BRD pathogens with concordance-based methods. The inconsistent Se of metagenomics for predicting susceptibility to different antimicrobials aligns with previous reports of variable agreement between AST and whole-genome sequencing on the Illumina platform [13]. Absolute agreement was strong in both previous studies between resistant to tetracycline and detection of the *tet(H)* gene. However, agreement was lower between detected genes and tulathromycin and substantially lower with tilmicosin [13]. Although these studies provided valuable comparisons between genotypic and phenotypic results, reported concordance analyses relied on calculation of absolute agreement statistics that did not account for expected agreement by chance. The use of BLCMs in the present study extends this prior work by generating Se and Sp estimates without assuming a gold standard, generating results that can be applied more directly to inform clinical interpretation under varying resistance prevalence scenarios using PPV and NPV.

Others have reported incomplete knowledge of resistance mechanisms and ARGs as an explanation for discordance between phenotypic and genotypic assessment of resistance [42]. Accurate prediction of resistance from genetic data relies on comprehensive databases, such as CARD [43], ResFinder [44], and MEGARes [45], which are cataloged as known resistance genes. However, new resistance genes continue to emerge. The recent characterization of the ARG *EstT* in *M. haemolytica* isolates recovered from BRD cases [46] is an important example. *EstT* was not included in earlier databases and might have accounted for some unexplained tilmicosin resistance observed in previous studies [13,14]. In another study, macrolide resistance was conferred by 23S rRNA mutations rather than specific resistance genes [47]. An advantage of sequencing is that the existing data can be re-analyzed to identify newly identified genes or resistance mechanisms, particularly given the relatively automated structure of the pipeline used in the present study and available computing capacity.

Further complicating the comparison of genotype and phenotype, resistance often results from interactions among multiple genes and regulatory elements [42]. The detection of an ARG does not guarantee its expression. Regulatory mechanisms, environmental factors, or laboratory conditions could suppress gene expression and lead to phenotypic susceptibility despite the presence of an ARG. However, in our study, diagnostic Sp for both AST and metagenomic sequencing were high for macrolides but slightly lower for tetracyclines in one of the two data sets, suggesting most of the detected target genes were expressed. However, diagnostic Se for metagenomics was more variable depending on the resistance pattern and population, suggesting undetected or unknown resistance mechanisms.

The metagenomic sequencing workflow of this study was designed to optimize Se and the capacity to link ARGs to bacteria of interest. Samples underwent bacterial enrichment prior to metagenomic sequencing to increase the abundance of *Pasteurellaceae* DNA and improve ARG detection [7,15]. Long-read metagenomic sequencing allowed for taxonomic assignment of ARGs directly to speciated *Pasteurellaceae* reads [7]. This is in contrast to short-read metagenomic sequencing, where reads are typically 150–300 bp long and often cannot capture both the ARG and sufficient flanking sequence to confidently assign the gene to a specific bacterial host [48].

While bacterial enrichment introduces certain limitations to metagenomic sequencing—such as potential biases in microbial community composition and added complexity in sample preparation—it remains a valuable enhancement to the sequencing protocol, particularly for diagnostic applications [7]. Enrichment can inadvertently favor fast-growing or nutrient-adapted taxa, which may distort the relative abundance of organisms and reduce the accuracy of broader microbiome profiling. However, its ability to increase sensitivity for detecting specific pathogens and ARGs, especially in samples dominated by host DNA, makes it a powerful tool for targeted investigations. Although it adds time and logistical demands to the workflow, enrichment significantly improves the resolution and reliability of pathogen detection, aligning well with clinical and diagnostic goals such as identifying BRD agents and associated ARGs. Therefore, while not ideal for comprehensive ecological studies, enrichment offers substantial benefits when the primary objective is focused microbial detection.

This study focused on AMR detection in three of the primary *Pasteurellaceae* species most often implicated in BRD: *M. haemolytica*, *P. multocida*, and *H. somni* [49,50]. Resistance was evaluated if present in any of the three organisms because in practice, BRD is managed as a syndrome, and antimicrobial treatment decisions are typically directed at the clinical signs of the animal, rather than a specific organism. These bacteria were assessed for resistance to macrolides and tetracyclines due to their importance for BRD management [9,51,52,53], known ARGs associated with resistance [54,55,56], and relative prevalence of specific AMR phenotypes within this population of cattle [29]. This allowed for this targeted comparison of metagenomics, culture and AST in these BRD bacteria of interest to provide guidance for future application of metagenomics to BRD testing, surveillance, and for antimicrobial stewardship decisions.

The ARGs most commonly identified on reads of respiratory bacteria and most associated with clinically relevant AMR in these samples were *tet(H)*, *msrE-mphE*, and *EstT.* These findings were consistent with other genomic studies of respiratory bacteria in beef cattle [14,20,56,57]. While other macrolide or tetracycline-associated ARGs, including *Erm(42)* and *tet(B)*, have been reported in the literature [13,14,58,59,60,61], they were observed in less than 1% of samples in the current study. Notably, *tet(H)* has shown consistent association with tetracycline resistance across previous reports [13,14,18,57,62,63] and in the present study. While *Erm(42)* and *msrE-mphE* have been the most commonly examined macrolide resistance genes in BRD pathogens, either together [14,59,64] or *Erm42* alone [13], the present study is relatively unique in examining *EstT* due to its more recent characterization [46].

Other ARGs were also detected in the metagenomic sequencing data, including those associated with sulfonamide (*sul2*) and aminoglycoside resistance (*APH(3″)-Ib*, *APH(3′)-Ia*, and *APH(6)-Id*). However, since aminoglycosides are not used in feedlot cattle [9], they were not included in this analysis. Furthermore, sulfonamides are typically used in combination with trimethoprim, for which there was little to no evidence of either ARGs or phenotypic resistance. In addition, there were no reported CLSI breakpoints for sulfonamides or the aminoglycosides necessary to classify resistance status for BLCM [29].

The genes *msrE* and *mphE* were evaluated due to their well-established role in conferring resistance to macrolides [59,64,65] and importance in BRD-associated bacteria, particularly *M. haemolytica* [66]. Together, these two genes encode efflux pumps and enzymatic mechanisms that reduce the effectiveness of macrolides by actively transporting the drug out of bacterial cells *(msrE)* and inactivating the drug *(mphE).* Additionally, *msrE* and *mphE* are typically arranged in tandem and co-expressed by the same promoter [60,64]. As such, these genes were grouped together for analysis. Occasional instances where these ARGs were detected separately were attributed to sequencing artifacts from fragmented reads.

The *EstT* gene was included in this analysis due to its role as an emerging resistance determinant [46]. *EstT* encodes a serine-dependent macrolide esterase, which hydrolyzes and inactivates macrolides, which was most effective at disrupting 16-membered ring macrolides such as tylosin, tilmicosin, and tildipirosin [46]. The 15-membered ring macrolides, tulathromycin and gamithromycin, were not affected by *EstT.* However, a recent investigation by Deschner et al. in 2024 using whole-genome sequencing of *M. haemolytica* isolates found that in isolates with resistance to gamithromycin, tulathromycin, tilmicosin, and tildipirosin, *EstT* was detected without the presence of other macrolide genes that could explain the 15-membered ring macrolide resistance [67]. Given the absence of consistent evidence regarding the associations between *msrE-mphE* or *EstT* and resistance to specific macrolides, this study evaluated the detection of these genes alongside AST results across multiple phenotypic resistance combinations. This comprehensive approach allowed for a more robust assessment of the diagnostic performance of metagenomic detection of each of these ARGs relative to varied phenotypic susceptibility outcomes.

Both AST and long-read metagenomics demonstrated high Sp for detection of antimicrobial non-susceptibility; Sp estimates were 97% and 98% for metagenomic sequencing identifying *msrE-mphE* or *EstT*. The resulting Sp estimates supported the decision to optimize Se and to set the cutpoints for classifying samples as positive if at least one bacterial read from a target species was identified with an ARG of interest. The high estimates of Sp were also consistent with previous reports supporting the relationship between *msrE-mphE* and phenotypically expressed resistance to 15-membered ring macrolides or *EstT* to 16-membered ring macrolides [46,64,67].

Temporal patterns in *Pasteurellaceae* detection using long-read metagenomic sequencing were broadly consistent with culture-based findings from the same population [29]. In 2020, detection of *M. haemolytica* by metagenomic sequencing increased across all time points, as was observed by culture [29]. Similarly, for both metagenomic sequencing and culture, the likelihood of *P. multocida* detection decreased from 1 DOF to 13 DOF, following metaphylaxis administration, before increasing again by 36 DOF. Similar directional changes were observed in 2021 for all organisms detected by both methods, with most having a lower likelihood of detection from 1 DOF to 13 DOF before increasing again over time. The marked increase in metagenomic detection of *H. somni* from 1 DOF to 36 DOF in 2021 oxytetracycline-treated cohorts was also consistent with a documented outbreak of histophilosis in one pen [29].

Similarly, metagenomic detection of bacteria with ARGs also mirrored phenotypic resistance trends across time points and metaphylaxis groups. In tulathromycin-treated cohorts, detection of the macrolide resistance genes *msrE-mphE* and *EstT* increased over time, consistent with observed higher likelihood of phenotypic non-susceptibility to 15- and 16-membered macrolides as reported for AST [29]. This pattern was particularly pronounced in 2020 tulathromycin-treated calves, where the prevalence of macrolide-resistant *M. haemolytica* increased over time for both metagenomic sequencing and AST results. The association between increasing detection of *M. haemolytica* with *mphE-msrE* and rising phenotypic resistance to 15-membered ring macrolides [29] align with the previously documented inter-pen spread of macrolide-resistant *M. haemolytica* in 6 of 8 pens from this population [68]. Likewise, in oxytetracycline-treated calves, *tet(H)* detection also increased over time, paralleling phenotypic tetracycline resistance patterns. The alignment of the longitudinal trends in metagenomic sequencing with that of culture-based results highlights the potential utility of using metagenomics for monitoring temporal trends in bacteria and AMR.

Bayesian latent class models have been previously applied to compare diagnostic accuracy of respiratory scoring systems for detecting clinical bovine respiratory disease [69,70,71,72]. A recent study compared whole genome sequencing to culture-based AST for assessing antimicrobial resistance in *Escherichia coli* isolates from cattle [73]. Another study by Bokma et al. in 2021, applied BLCMs to evaluate the diagnostic accuracy of nanopore sequencing for identifying *M. bovis* in bronchoalveolar lavage fluid, showing high Sp and moderate Se compared to real-time PCR and MALDI-TOF [74]. Benedict et al. used BLCMs in 2014 to assess the accuracy of AST by disk diffusion and broth microdilution by testing isolates of *E. coli* and *M. haemolytica* for susceptibility to ampicillin, ceftiofur, streptomycin, sulfisoxazole, tetracycline, and trimethoprim-sulfamethoxazole [62]. However, no prior studies have applied BLCMs to estimate the Se and Sp of metagenomics and culture for detecting multiple BRD bacteria and associated AMR and ARGs. This study, therefore, represents a novel application of BLCM to characterize the diagnostic performance of metagenomic sequencing and AST for resistance detection in BRD bacteria.

In this study, the latent class was defined as “potential for resistance,” aiming to explore the utility of culture and AST versus metagenomic sequencing in estimating the broader biological potential for antimicrobial resistance or transmission potential as represented for the sample. Neither test provides a definitive prediction of in vivo resistance to treatment. Antimicrobial susceptibility testing measures the response of a bacterium to antimicrobials under controlled laboratory conditions and does not necessarily predict clinical outcomes in vivo [75,76,77,78]. Further, AST results are limited by the number of isolates that can be tested per species per sample. Similarly, while metagenomic sequencing identifies ARGs, their presence alone is a sign of functional potential not an indicator of gene expression, nor is the presence of the ARG necessarily linked to clinical outcomes.

Although this study included metagenomic sequencing results from a large number of samples, the precision of the analysis was constrained by the prevalence of phenotypic resistance [29,79] and ARGs of interest present within the population of calves sampled. The limited number of ARG-positive samples resulted in the wide CrI observed, specifically for *EstT*, and precluded the evaluation of macrolide resistance related to *mphE-msrE* in the 2021 cohort.

While this research group found that the metagenomic sequencing protocol was sensitive enough to detect *Pasteurellaceae* of interest from the DNP samples in feedlot calves [15], a non-specific enrichment step was required to optimize bacterial DNA quantity and to obtain sufficiently long DNA fragments to assign ARGs to specific bacterial reads [7]. This is an important consideration to note as one of the assumptions of latent class models is that of conditional independence between the laboratory tests being compared [80,81]. There were no substantial differences in the Se and Sp estimates in models that included covariance terms to measure and account any lack of independence between culture and metagenomics compared to those models that did not.

Significant differences in bacterial and ARG prevalence across years, timepoints and metaphylaxis groups support another key BLCM assumption that the defined populations exhibit distinct prevalences. A final assumption required for application of BLCMs is that the tests exhibit constant Se and Sp across populations. While this assumption is challenging to formally assess, a supplemental analysis in which the arrival population was dropped from the analysis produced minimal changes in model estimates, providing support that this assumption was reasonable in this study.

The DNPS collected and utilized in this study resulted from planned samples from calves not showing signs of clinical BRD. The metagenomic sequencing performance could be different in animals with clinical BRD that could be shedding a greater pathogen load [82,83]. Phenotypic AMR and ARGs would be expected to vary across animals that had received different antimicrobials for metaphylaxis or multiple antimicrobial treatments [29,79,84,85,86,87,88]. Therefore, further investigations into the utility of metagenomic sequencing as a laboratory tool for BRD and AMR in cattle with clinical disease are warranted.

## 4. Methods

### 4.1. Ethics Statement

This study was conducted in accordance with the recommendations of the Canadian Council of Animal Care (CCAC) [89]. The research protocols and procedures for this study were approved by the University of Saskatchewan Animal Care Committee (AUP 20190069) on 30 May 2019 and reviewed annually until completion.

### 4.2. Animals and Sample Population

Recently weaned, mixed-breed steer calves were purchased from a western Canadian auction market during the fall of 2020 (*n* = 800) and 2021 (*n* = 800) and transported to a research feedlot operated by the University of Saskatchewan. A detailed summary of calf characteristics, animal housing and management was previously described [29]. Auction-sourced calves were purchased in lots of 100 and housed in adjacent, outdoor earthen floor pens with their arrival cohort for the duration of the study period. Calves were provided a diet formulated to meet or exceed the National Research Council nutritional requirements for beef cattle [90].

All calves were processed at arrival following standard industry practices, including individual tags, vaccinations, parasite control, castration verification, and injectable metaphylaxis. In the fall of 2020, all calves (*n* = 800) received metaphylactic tulathromycin as a single dose of 2.5 mg/kg of body weight (BW) (Draxxin^®^, Zoetis Inc., Florham Park, NJ, USA). In the fall of 2021, half of the pens (*n* = 400 calves) were administered metaphylactic tulathromycin, while the other half (*n* = 400 calves) were administered oxytetracycline (Oxyvet^®^200 LA, Vetoquinol, Lavaltrie, QC, Canada) subcutaneously as a single dose of 20 mg/kg of BW.

The average BW of calves at arrival processing in 2020 was 253 kg (range 211–291 kg), and in 2021 was 225 kg (range 351–694 kg) [29]. Herd of origin was approximated using the first 12 digits of the 15-digit RFID tags required for all cattle moved between premises. The number of unique herds of origin for 2020 calves was 291 and ranged from 30 to 81 herds per pen, while the calves sourced in 2021 were slightly less diverse, with 208 unique herds of origin and 12–38 unique herds per pen [29].

**Figure 2:**
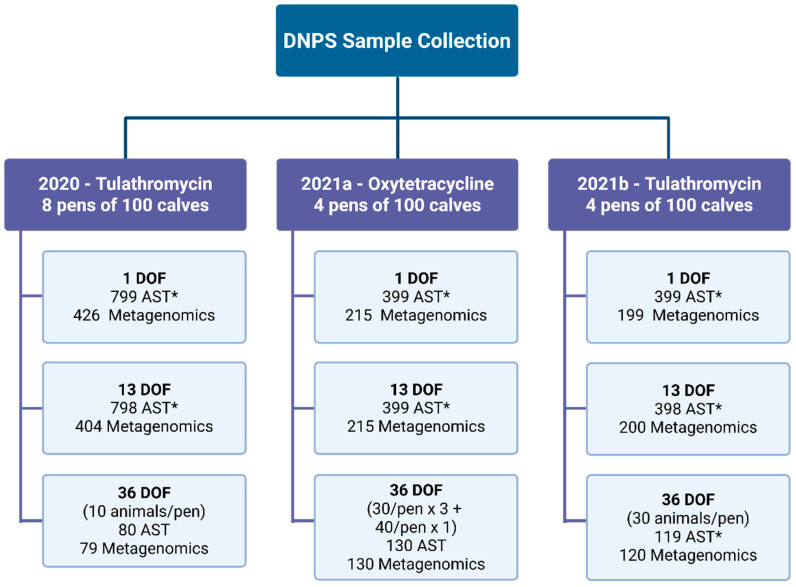
Deep nasopharyngeal swab (DNPS) sample collection and testing numbers by method, year, metaphylaxis type (tulathromycin or oxytetracycline), and time point (DOF—days on feed). * Sample sizes tested by antimicrobial susceptibility testing (AST) < 100 calves per pen were due to animal mortality or missing AST data.

Details regarding the procedure for collecting deep nasal pharyngeal swabs (DNPS) were previously reported [29] and were summarized in Figure 2. One animal was removed due to lameness, and the remaining 1599 calves were sampled at two time points: at arrival processing (1 day on feed) prior to antimicrobial metaphylaxis administration and at 13 days on feed (13 DOF). A random subset of calves from each pen was also sampled at 36 DOF (10 per pen in 2020 and 30 per pen in 2021) [29]. Samples were collected from calves secured in a hydraulic chute with a neck extender. Three DNPS were collected from alternating nostrils of each calf at each sampling time and combined into a single pooled vial containing 3 mL of liquid Amies transport media (CoPan Diagnostics, Carlsbad, CA, USA). Aliquots from the pooled vials were used for subsequent bacterial culture and AST as well as metagenomic sequencing.

### 4.3. Bacteriology and AST Protocols

All culture and AST protocols were previously described elsewhere [29]. Samples were transported on ice to the University of Saskatchewan for same-day processing. The samples were vortexed for 1 min and a 300 μL aliquot was submitted to Prairie Diagnostic Services, Inc. (Saskatoon, SK, Canada, PDS) for same-day batch processing. For *M. haemolytica*, *P. multocida*, and *H. somni* cultures, a 10 μL inoculation loop of sample was cultured on Columbia agar with 5% sheep blood (BA), and a second loop was cultured on chocolate agar (CHOC) (Oxoid, Thermo Fisher, Waltham, MA, USA); plates were incubated at 35 °C for 18 h in 5% CO_2_. One isolate exhibiting phenotypic morphologies for each bacterium of interest was selected and speciation confirmed using MALDI-TOF MS (Bruker Daltonik, Bremen, Germany), according to manufacturer guidelines and Biotyper Microflex LT Compass software (version 1.4). Only colonies with MALDI-TOF MS identification scores ≥ 2.0 were included in further analysis. Positive and negative controls were tested on all sample processing days and new media batches. The positive controls were *Staphylococcus aureus* ATCC 29213, *Escherichia coli* ATCC 25922, and *Histophilus somni* ATCC 700025.

One colony from each target species was selected from the purity plates for AST using a commercially available bovine serial broth microdilution panel (Thermo Fisher Scientific^TM^, Waltham, MA, USA, Bovine AST BOPO7F Plate) on the Sensititre^TM^ platform. The minimum inhibitory concentration (MIC) plate was placed and read on the BIOMIC^®^ V3 microplate reader. Quality control measures were performed according to the manufacturer’s guidelines using *E. coli* ATCC 25922, *S. aureus* ATCC 29213, and *H. somni* ATCC 700025. The MIC value was considered equal to the lowest concentration of antimicrobial that inhibited visible growth. The MIC for each antimicrobial was compared to the Clinical and Laboratory Standards Institute (CLSI) breakpoints [91], except for tilmicosin, for which breakpoints are only available for *M. haemolytica* [29].

### 4.4. Metagenomic Sequencing Sample Preparation Protocols

A subset of collected samples was selected for metagenomic sequencing to allow for sequencing at least half of the samples from each pen. Initially, all calves that received treatment for BRD or from the subset of calves sampled at 36 DOF were identified. They and their matching samples from arrival processing and 13 DOF were selected for sequencing from each pen. Additional calf samples from 13 DOF with matching samples at 1 DOF arrival were then randomly selected as necessary to provide sequence samples from a total of at least 50 calves per pen.

### 4.5. Sample Processing Protocol

The DNPS collected in the fall of 2020 were stored at −80 °C and then processed for metagenomic sequencing using a protocol modified from one optimized through development work in 2021 [7].

A sample enrichment step was completed before DNA extraction. The swab heads were placed into glass vials with stir bars capped with air-permeable membranes (Thomson, Carlsbad, CA, USA) within 7–10 mL of brain heart infusion broth (BHI) (Oxoid, Thermo Fisher, Waltham, MA, USA) + 1% glucose. Vials were aerated and incubated at 35–37 °C for 14 h. Enriched media was collected for downstream DNA extraction.

A 1.5 mL aliquot of each enriched sample was pelleted at 4000 rpm × 10 min and then resuspended in 100 μL 1× phosphate-buffered saline (PBS). For host depletion, the sample was treated with 1× DNase (Invitrogen, Waltham, MA, USA) for 30 min at 37 °C × 300 rpm. DNase was inactivated via heat treatment at 75 °C for 10 min × 300 rpm. Samples were pelleted by centrifugation at 4000 rpm × 10 min and washed by resuspension in 100 μL with 1× PBS to remove DNase. After removing the wash buffer, cells were again pelleted by centrifugation at 4000 rpm × 10 min to prepare for extraction.

DNA extraction was completed via alcohol precipitation, using the Qiagen Puregene Buccal Cell kit (QIAGEN Inc., Germantown, MD, USA). Pelleted cells were washed and resuspended in Qiagen Puregene Cell Lysis buffer (QIAGEN Inc., Germantown, MD, USA), then treated with proteinase K (QIAGEN Inc., Germantown, MD, USA) at 55 °C for 1 h at 300 rpm. Following protein digestion, samples were treated with RNase A (QIAGEN Inc., Germantown, MD, USA) at 37 °C for 15 min at 300 rpm. Liberated proteins were precipitated via Qiagen Puregene Protein Precipitation solution (QIAGEN Inc., Germantown, MD, USA) and then cooled on ice until proteins had coagulated and could be pelleted by centrifugation.

Supernatants were carefully removed to fresh tubes to avoid protein contamination. Nucleic acids were precipitated using isopropanol with glycogen (QIAGEN Inc., Germantown, MD, USA) as a carrier, incubated at room temperature for 5 min to ensure maximum DNA retention, then spun for 5 min at max speed (14,000 to 16,000 rpm). Pellets were then washed with 70% (*v*/*v*) ethanol. The ethanol wash was removed, and the pellet was allowed to air dry. The DNA pellet was resuspended in Qiagen Puregene DNA Hydration solution (QIAGEN Inc., Germantown, MD, USA) for 1 h at 65 °C and then overnight by gentle rotation at room temperature. Host-depleted, extracted DNA was stored at 4 °C until sequencing.

A minimum of 2 μg DNA (50 ng/μL in 40 μL) was used for library preparation. Samples were tested using Qubit 1× dsDNA HS Assay kit (Invitrogen, Waltham, MA, USA), and 394 of the samples were re-extracted from the reserve enrichment using additional aliquots to obtain the required 2 μg DNA.

The DNPS collected in the fall of 2021 were processed immediately after collection. Swabs and remaining media were incubated in BHI + 1% glucose at 35–37 °C as described for the 2020 samples, but for 10 h (Supplementary Table S1). DNase treatment was not performed on the 2021 samples, as pilot testing by this group indicated it was not necessary to achieve adequate metagenomic sequencing results on samples processed without prior freezing [7]. Sample DNA was extracted and processed as for the 2020 samples. Extracted DNA was stored at 4 °C until sequencing.

### 4.6. Library Preparation and Metagenomic Sequencing Protocol

The 2021 samples prepared from fresh swabs and DNA were sequenced first, followed by the 2020 samples that were prepared from frozen swabs (Table S1). Extracted DNA was purified and size selected by a 0.4× clean up with AMPure XP beads (Beckman Coulter, Indianapolis, IN, USA). Normalization of samples to 400 ng was performed (where sufficient DNA was present, or 200 ng if not) prior to library preparation. For the samples with insufficient DNA concentration after size selection, non-size-selected DNA was used instead.

Library preparation was completed following Oxford Nanopore Technologies (ONT) ligation protocol with native barcoding (SQK-LSK109 and EXP-NBD196, Oxford Nanopore Technologies, Oxford, UK) in a 96-well plate high-throughput library format with minor modifications to minimize barcode crossover or background barcode crosstalk [92]. In brief, barcode ligation was followed by the addition of 1 µL of EDTA (Invitrogen, Waltham, MA, USA), a 10 min room temperature incubation and a 10 min 65 °C incubation. Barcoded DNA from samples was pooled into 36 libraries, with each library containing up to three negative controls (nuclease-free water, Invitrogen, Waltham, MA, USA).

From each prepared library, 80–200 ng (2021 samples) and 70–150 ng (2020 samples) (Table S1) were sequenced by the Omics and Precision Agriculture Laboratory (OPAL, University of Saskatchewan) with the PromethION24 platform. Flow cells (FLO-PRO002, ONT version R9.4.1, Oxford Nanopore Technologies, Oxford, UK) were loaded according to the manufacturer’s instructions, except for using molecular-grade H_2_O (Sigma-Aldrich, St. Louis, MO, USA or Promega, Madison, WI, USA) in place of loading beads to reduce pore saturation during sequencing.

Metagenomic sequencing was carried out with standard default run parameters using high-accuracy base calling with a cut-off of 7. Sequencing runs were active for 72 h for the 2021 samples and 48 h for the 2020 samples (Table S1). The change from 72 h (2021 samples) to 48 h (2020 samples) followed a series of retrospective analyses on 2021 sequence data to assess the rates of data acquisition and identified that sequencing beyond 48 h did not contribute to a different interpretation of a sample.

### 4.7. Preprocessing/Quality Control (QC)

Data from MinKNOW were processed for quality control with Porechop (version 0.2.4) [93] and NanoFilt (version 2.8.0) [94] to remove adapters and short (<200 bp) reads. NanoStat (version 1.6.0) provided statistics about the distribution of read length by total base pairs per sample [94].

### 4.8. Read Classification and Host Filtering

The taxonomic classification of reads was achieved using Kraken 2 (version 2.1.2) [95]. A custom database was used for Kraken 2 classification, which included bacterial, viral, and archaeal subsets of the November 2023 RefSeq database [96] as well as the *Bos taurus* ARS-UCD1.2_Btau5.0.1Y genome assembly available at https://sites.ualberta.ca/~stothard/1000_bull_genomes/ (accessed on 26 October 2025) [15,97]. Typically, sequences classified as host would be removed before downstream processing; however, a small population of chimeric *B. taurus* bacterial reads (<0.1% of all reads) was detected. A custom program, kmer_filter.py, was written to retrieve host-classified reads that met a threshold of 25% non-host sequence using Kraken 2 k-mer identity and include these reads for downstream processing. The rationale behind this step was to cast the widest possible net for ARG detection, even if a small amount of host sequence remained. Host-filtered reads (i.e., those not similar to the *B. taurus* taxid 9913) were extracted using the KrakenTools version 1.2 [98] utility extract_kraken_reads.py, and these were added to the chimeric reads using a combination of bash utilities and the BBTools (version 38.86) [99] filterbyname.sh script. Bracken (version 2.7) [100] with a minimum read length of 200 bp (“--read-length 200”) was used to improve the species-level estimation of abundance reported by Kraken 2. Reads classified as host were removed from further consideration. A custom script, report_taxon_read_lengths.py, added additional context to the Bracken results, including the total amount of sequence in base pairs reported for each species (including child taxa) and the fraction of total classified sequence.

### 4.9. Antimicrobial Resistance Gene Detection

To identify reads containing ARGs, non-host reads were converted to FASTA format using Seqtk (version 1.3) [101]. ARGs were identified in non-host reads based on Abricate (version 1.0.0) [102] and AMRFinderPlus (version 3.11.18) [103], analysis with the NCBI Bacterial Antimicrobial Resistance Reference Gene Database (version 2023-11-15.1). Abricate was also run using the Comprehensive Antimicrobial Resistance Database (CARD) (version 3.2.8) [104]. For AMRFinderPlus, the minimum percent identity and percent coverage thresholds were set to 80% and the -plus option was used to direct the program to search for genes involved in virulence, biocide, heat, metal, and acid resistance. Default parameters were used for Abricate (80% minimum percent identity and percent coverage). ARG results from the NCBI and CARD were merged based on gene name and start/stop coordinates. Once merged, the CARD gene names were preferentially used in downstream reports.

All pipeline outputs of interest were summarized for each sample as appropriate in customized series of Excel spreadsheets available to the study team on a custom-built portal that allowed for uploading and linking sample metadata.

### 4.10. Data Management and Statistical Analyses

Calf data, culture, AST, summary taxonomy, and ARG results were managed in a spreadsheet (Microsoft Excel, version 2401, Microsoft Corporation, Redmond, WA, USA). Descriptive analyses were completed using a commercial statistical software package (Stata/IC, version 16.1, StataCorp LLC, College Station, TX, USA).

The median of total read length in base pairs per sample, sample median read length, sample theoretical coverage, and number of reads for each of *M. haemolytica*, *P. multocida*, and *H. somni* were summarized across all samples from 2020 and 2021. Additionally, the number of samples in which any *msrE* and/or *mphE*, *EstT* or *tet(H)* reads were detected within *M. haemolytica, P. multocida* and/or *H. somni* reads were also summarized by year, metaphylaxis type and time of sampling. Similarly, all AST results for the antimicrobials considered in the BLCM were summarized across *M. haemolytica, P. multocida* and/or *H. somni* isolates from each sample. For the purposes of the primary BLCM analysis, based on available CLSI breakpoints for specific drug and bacteria combinations, to obtain a binary outcome, intermediate MIC values were categorized as resistant and reported as non-susceptible [91].

### 4.11. Bayesian Latent Class Models

Bayesian latent class models were developed and run using JAGS software v4.3.0 [105] and the runjags package [106] in R v4.1.0 [107]. Uninformative priors [beta(1,1)] were used for Se, Sp, and prevalence for each population. Convergence was evaluated using diagnostics, including potential scale reduction factor (<1.05), effective sample size (>1000), and Monte Carlo standard errors as a percent of standard deviation (<5%), as well as by visual inspection of trace and autocorrelation plots and was deemed satisfactory. Results for Se and Sp were reported as medians of the posterior distributions along with 95% credible intervals (CrIs). Predicted true prevalence was not reported as the subset chosen for metagenomic sequencing was not intended to be representative of the target population.

Two-test (AST vs. long-read metagenomic sequencing), three-population BLCMs were used for the 2020 analysis, while two-test, five-population models were used for 2021. For the 2020 analyses where all calves received tulathromycin as metaphylaxis, results from all samples at arrival processing (prior to metaphylaxis), 13 DOF, and 36 DOF were considered as different populations. In 2021, half of the pens received tulathromycin and half received oxytetracycline for metaphylaxis. Therefore, five populations were considered for 2021, defined as the DNPS samples collected from all calves at arrival processing (pre-metaphylaxis administration), calves receiving tulathromycin sampled at 13 DOF or 36 DOF, and calves receiving oxytetracycline sampled at 13 DOF or 36 DOF.

Initially, Bayesian latent class models were developed to compare the diagnostic test performance of culture and long-read metagenomic sequencing for the detection of *M. haemolytica, P. multocida*, and *H. somni*. The latent class for these models was defined as the detection of the organism in DNP samples. The classification of a sample as positive or negative by metagenomic sequencing was based on theoretical coverage. Theoretical coverage for each sample was calculated by dividing the total read length for each bacterium of interest by the size of its reference genome (*M. haemolytica*: 2.8 Mb [NCBI GCF_002285575.1], *P. multocida*: 2.3 Mb [NCBI CF_002073255.2], *H. somni*: 2.3 Mb [NCBI GCF_000019405.1]). Theoretical coverage was then evaluated against culture results using receiver operating characteristic curves in R [107] to determine a baseline cut-off maximizing combined Se and Sp (Youden’s index) of metagenomics compared to culture. This baseline cut-off was used for an initial classification of samples for comparison to culture using BLCM. The minimum threshold for Sp from BLCM of metagenomics was 0.90. If the initial BLCM Sp estimate was <0.90, the theoretical coverage cut-off was sequentially increased by increments of 50% and the BLCM was repeated until a Sp of ≥0.90 was obtained. Estimates for Se and Sp were compared among testing options and years and considered significantly different if the 95% credible interval (95% CrI) did not overlap. The resultant cut-off was then used to classify metagenomic sequencing data as positive or negative for the final models.

Latent class models assume conditional independence between laboratory tests. While culture and metagenomic sequencing are profoundly different processes, the bacterial growth enrichment step that occurred prior to DNA extraction for metagenomic sequencing could potentially introduce dependence between culture and sequencing. To assess the impact of potential dependence on BLCM estimates, a sensitivity analysis was conducted by constructing secondary BLCMs with covariance terms between test Se and Sp. Results were compared to the primary BLCM without the covariance terms and reported in the supplementary materials.

Further BLCMs were developed to evaluate Se and Sp for the detection of *M. haemolytica*, *P. multocida*, or *H. somni* with phenotypic non-susceptibility as determined by AST to specific antimicrobials, as compared to the detection of ARGs considered important determinants of corresponding AMR for that drug class in BRD-associated bacteria. In these models, the latent class was defined as the potential for AMR that could influence treatment outcomes and transmission of resistant organisms or genes. Targets for modeling were based on the prevalence of AST and ARGs, reported CLSI breakpoints to classify AST results, as well as the potential clinical importance of the AMR to BRD management. The resulting models compared phenotypic non-susceptibility based on AST to: (1) any macrolide (gamithromycin, tulathromycin, tildipirosin, or tilmicosin) with detection of *msrE-mphE* or *EstT*, (2) gamithromycin or tulathromycin (15-membered ring macrolides) and the detection of *msrE-mphE* or *EstT*, (3) tildipirosin or tilmicosin (16-membered macrolides) with detection of *msrE-mphE* or *EstT* and (4) tetracycline with detection of *tet(H).*

For AST, positive test results were defined as the isolation of *M. haemolytica*, *P. multocida*, or *H. somni* from DNPS (identified via MALDI-TOF MS) collected from individual calves, followed by classification of the isolates as intermediate or resistant (non-susceptible) to the target macrolides or to tetracycline. For long-read metagenomic sequencing, a positive result was defined as a sample where there was detection of the ARG of interest on any read bioinformatically identified as *M. haemolytica*, *P. multocida* or *H. somni*. The identification of *msrE* and *mphE* was considered in combination and defined as “either” or “both,” since these genes are typically arranged in tandem and co-expressed from the same promoter [64]. Models were developed only if there were at least five total samples with phenotypic non-susceptibility and detection of the ARG of interest.

To assess the impact of considering intermediate AST results as resistant (non-susceptible) versus non-resistant, the models comparing AST with metagenomic detection of ARG were also run with the AST intermediate results classified as susceptible instead of resistant. Results of these models were also reported as a sensitivity analysis in the supplementary materials.

Finally, models were constructed to assess the sensitivity of models for bacteria and AMR/ARG detection to the assumption that test performance was consistent among populations. Because the prevalence of the BRD bacteria and AMR determinants was expected to be considerably different in the on-arrival (1 DOF) samples compared to the other time points, based on previously documented phenotype data [29], models were run excluding this population (1 DOF). Estimates were compared to the full models, and results were reported in supplementary materials.

Estimates of positive and negative predictive value (PPV, NPV) were generated for key test combinations across a range of possible pre-test probabilities for detection of both respiratory bacteria and AMR [108].

### 4.12. Statistical Analysis of Differences in Bacterial ARG Detection Among Time Points

For differences over time in the phenotypic results, full details on the statistical models have been reported [29]. In the current analyses focusing on the metagenomic sequencing data, differences in each outcome of interest across sampling time points (arrival processing (1 DOF), 13 DOF, and 36 DOF) were analyzed using three-level mixed-effects logistic regression models (Stata/IC, version 16.1, StataCorp LLC, College Station, TX, USA). Fixed effects included sampling time point and a combined term accounting for both study population year and the metaphylactic antimicrobial used. Repeated measures on individual calves and calves nested within pens were described with random intercepts. When mixed-effects models failed to converge due to sampling time points with few or zero positive calves, exact logistic regression models were used to generate estimates.

## 5. Conclusions

The application of BLCMs for BRD pathogens and associated ARGs in samples from feedlot calves establishes a baseline understanding of the performance of long-read metagenomic sequencing relative to culture and AST. Overall, Sp was good to very good for most targets for both culture/AST and metagenomics, but Se was more variable. The results from this work highlight the strengths and limitations of long-read metagenomic sequencing, providing valuable insights for future refinement and potential adoption. Estimates of Se and Sp for key outcomes were sufficiently comparable to motivate additional research examining the clinical relevance of metagenomic detection of bacteria and ARGs for informing antimicrobial stewardship. Furthermore, this study serves as a foundational assessment, allowing for expansion to a broader range of pathogens, diverse sample populations, and varied diagnostic contexts. By building on this baseline, future efforts can further enhance our understanding and application of these diagnostic tools.

## Figures and Tables

**Table 1:** Summary of long-read metagenomic sequence statistics for *M. haemolytica*, *P. multocida*, and *H. somni* in deep-nasopharyngeal swab samples from feedlot calves in 2020 and 2021, where corresponding culture data were available for use in Bayesian latent class models.

Year	Bacteria	Median Total Base Pairs (bp)	Median of Per Sample Median Read Lengths (bp)	Median Theoretical Coverage (×Genome Size) ^1^	Median Number of Reads	Percent (%) of Samples with ≥1 Read
2020 (*n* = 909 samples)	*M. haemolytica*	5,161,422	1321	1.8	2304	100% (909)
	*P. multocida*	1,088,197	1371	0.47	525	99.7% (906)
	*H. somni*	11,787	1099	0.01	8	89.5% (814)
2021 (*n* = 1079 samples)	*M. haemolytica*	3,979,840	3858	1.4	775	100% (1079)
	*P. multocida*	228,236	3351	0.10	46	99.3% (1071)
	*H. somni*	13,170	2591	0.01	4	78.3% (845)

^1^ Theoretical coverage for each sample was calculated by dividing the total read length for each of the bacteria of interest by the size of its reference genome (*M. haemolytica*: 2.8 Mb [NCBI GCF_002285575.1], *P. multocida*: 2.3 Mb [NCBI GCF_002073255.2], *H. somni*: 2.3 Mb [NCBI GCF_000019405.1]).

**Table 2:** Antimicrobial resistance genes (ARGs) most frequently detected by long-read metagenomic sequencing in reads identified as *M. haemolytica*, *P. multocida*, and/or *H. somni* from 2020 and 2021, along with the corresponding drug class resistance for each gene (*n* = 1985 samples) in samples with corresponding culture and AST data for use in Bayesian latent class models.

		Number (%) of Samples in Which Gene Was Detected		
Gene ^1^	Resistance class	Total (*n* = 1985)	2020 (*n* = 909)	2021 (*n* = 1076) ^2^
*sul2*	sulfonamides	327 (16%)	234 (26%)	93 (8.6%)
*tet(H)*	tetracyclines	313 (16%)	138 (15%)	175 (16%)
*mphE*	macrolides	181 (9.1%)	178 (20%)	3 (0.3%)
*msrE*	macrolides	177 (8.9%)	174 (19%)	3 (0.3%)
*APH(3″)-Ib*	aminoglycosides	165 (8.3%)	85 (9.4%)	80 (7.4%)
*APH(3′)-Ia*	aminoglycosides	160 (8.1%)	84 (9.2%)	76 (7.1%)
*APH(6)-Id*	aminoglycosides	157 (7.9%)	81 (8.9%)	76 (7.1%)
*EstT*	macrolides	131 (6.6%)	60 (6.6%)	71 (6.6%)

^1^ ARGs found in >100 samples. ^2^ Corresponding AST data were not available for 3 of 1079 samples.

**Table 3:** Frequency of samples with the antimicrobial resistance genes *msrE-mphE*, *EstT*, and/or *tet(H)* detected by long-read metagenomic sequencing on at least one read identified as *M. haemolytica*, *P. multocida*, or *H. somni* at arrival processing, 13, and 36 days on feed for use in Bayesian latent class models.

		2020 (*n* = 909)				2021 (*n* = 1076) ^1^							
		Tulathromycin Metaphylaxis				Oxytetracycline Metaphylaxis (*n* = 559)				Tulathromycin Metaphylaxis (*n* = 517)			
	DOF	*n*	*msrE-mphE*	*EstT*	*tet(H)*	*n*	*msrE-mphE*	*EstT*	*tet(H)*	*n*	*msrE-mphE*	*EstT*	*tet(H)*
*M.* *haemolytica*	Arrival processing	426	10 (2.3%)	5 (1.2%)	52 (12%)	214	0	2 (0.9%)	5 (2.3%)	198	0	0	7 (3.5%)
	13	404	134 (33%)	33 (8.2%)	57 (14%)	215	0	8 (3.7%)	45 (21%)	200	1 (0.5%)	27 (14%)	36 (18%)
	36	79	40 (51%)	11 (14%)	22 (28%)	130	0	2 (1.5%)	32 (25%)	119	1 (0.8%)	28 (24%)	34 (29%)
*P. multocida*	Arrival processing	426	2 (0.5%)	1 (0.2%)	22 (5.2%)	214	0	0	2 (0.9%)	198	0	0	5 (2.5%)
	13	404	9 (2.2%)	3 (0.7%)	15 (3.7%)	215	0	0	25 (12.0%)	200	0	0	8 (4.0%)
	36	79	0	0	1 (1.3%)	130	0	0	19 (15%)	119	0	0	6 (5.0%)
*H. somni*	Arrival processing	426	1 (0.2%)	6 (1.4%)	20 (4.7%)	214	0	0	0	198	0	0	0
	13	404	59 (15%)	30 (7.4%)	10 (2.5%)	215	0	4 (1.9%)	11 (5.1%)	200	0	15 (7.5%)	8 (4.0%)
	36	79	17 (22%)	6 (7.6%)	3 (3.8%)	130	0	1 (0.8%)	15 (12%)	119	2 (1.7%)	16 (13%)	12 (10%)
Any of: *M.* *haemolytica*, *P. multocida*, or *H. somni*	Arrival processing ^2^	426	13 (3.1%)	9 (2.1%)	58 (14%)	214	0	2 (0.9%)	5 (2.3%)	198	0	0	9 (4.5%)
	13	404	138 (34%)	39 (10%)	58 (14%)	215	0	8 (3.7%)	52 (24%)	200	1 (0.5%)	30 (15%)	36 (18%)
	36	79	40 (51%)	12 (15%)	22 (28%)	130	0	2 (1.5%)	37 (28%)	119	2 (1.7%)	29 (24%)	36 (30%)

DOF—days on feed. ^1^ Corresponding AST data were not available for 3 of 1079 samples. ^2^ The counts for 2021 at arrival processing samples for the different metaphylaxis groups were combined to form a single population for the Bayesian latent class modeling, as metaphylaxis was not administered until after arrival processing sampling, and there was, therefore, no expectation that prevalence of ARGs would differ between groups.

**Table 4:** Frequency of samples for which *M. haemolytica*, *P. multocida*, and/or *H. somni* isolates were classified as non-susceptible (resistant or intermediate per CLSI breakpoints) to selected macrolides or tetracycline by antimicrobial susceptibility testing (AST) at arrival processing, 13, and 36 days on feed for use in Bayesian latent class models ^1,2^.

		2020 (*n* = 909)					2021 (*n* = 1076) ^3^									
		Tulathromycin Metaphylaxis					Oxytetracycline Metaphylaxis (*n* = 559)					Tulathromycin Metaphylaxis (*n* = 517)				
	DOF	*n*	Any Macrolide	GAM or TULA	TILD or TILM ^4^	TET	*n*	Any Macrolide	GAM or TULA	TILD or TILM ^4^	TET	*n*	Any Macrolide	GAM or TULA	TILD or TILM ^4^	TET
*M.* *haemolytica*	Arrival processing	426	5 (1.2%)	1 (0.2%)	5 (1.2%)	5 (1.2%)	214	8 (3.7%)	0	8 (3.7%)	0	198	5 (2.5%)	0	5 (2.5%)	0
	13	404	196 (49%)	196 (49%)	135 (33%)	14 (3.5%)	215	4 (1.9%)	1 (0.5%)	4 (1.9%)	1 (0.5%)	200	22 (11%)	21 (11%)	22 (11%)	21 (11%)
	36	79	44 (56%)	43 (54%)	33 (42%)	8 (10%)	130	5 (3.8%)	0	5 (3.8%)	0	119	27 (23%)	10 (8.4%)	27 (23%)	26 (22%)
*P. multocida*	Arrival processing	426	0	0	0	13 (3.1%)	214	0	0	0	4 (1.9%)	198	0	0	0	4 (2.0%)
	13	404	1 (0.2%)	1 (0.2%)	0	5 (1.2%)	215	0	0	0	29 (13%)	200	0	0	0	0
	36	79	1 (1.3%)	1 (1.3%)	0	3 (3.8%)	130	0	0	0	19 (15%)	119	1 (0.8%)	1 (0.8%)	0	0
*H. somni*	Arrival processing	426	22 (5.2%)	22 (5.2%)	16 (3.8%)	0	214	2 (0.9%)	2 (0.9%)	0	1 (0.5%)	198	0	0	0	0
	13	404	8 (2.0%)	6 (1.5%)	2 (0.5%)	0	215	1 (0.5%)	0	1 (0.5%)	11 (5.1%)	200	1 (0.5%)	1 (0.5%)	0	0
	36	79	12 (15%)	12 (15%)	5 (6.3%)	0	130	2 (1.5%)	2 (1.5%)	0	12 (9.2%)	119	2 (1.7%)	2 (1.7%)	0	6 (5.0%)
Any of: *M. haemolytica*, *P. multocida*, or *H. somni*	Arrival processing	426	27 (6.3%)	23 (5.4%)	21 (4.9%)	18 (4.2%)	214	10 (4.7%)	2 (0.9%)	8 (3.7%)	5 (2.3%)	198	5 (2.5%)	0	5 (2.5%)	4 (2.0%)
	13	404	201 (50%)	200 (50%)	136 (34%)	19 (4.7%)	215	5 (2.3%)	1 (0.5%)	5 (2.3%)	40 (19%)	200	23 (12%)	22 (11%)	22 (11%)	21 (11%)
	36	79	53 (67%)	52 (66%)	36 (46%)	11 (14%)	130	7 (5.4%)	2 (1.5%)	5 (3.8%)	31 (24%)	119	30 (25%)	13 (11%)	27 (23%)	32 (27%)

DOF—days on feed; GAM—gamithromycin; TULA—tulathromycin; TILD—tildipirosin; TILM—tilmicosin; TET—tetracycline. ^1^ The counts for 2021 1 DOF samples for the different metaphylaxis groups were combined to form a single population for the Bayesian latent class modeling, as metaphylaxis was not administered until after arrival processing sampling and there was therefore no expectation that prevalence of ARGs would differ between groups. ^2^ CLSI breakpoints were not available to classify the corresponding MIC data for these organisms for sulphadimethoxine, trimethoprim-sulfamethoxazole, gentamicin or neomycin. ^3^ Corresponding AST data were not available for 3 of 1079 total samples. ^4^ CLSI breakpoints were not available to classify MICs for TILM for *P. multocida* and *H. somni* [1].

**Table 5:** Number (percent) of samples for positive detection of *M. haemolytica*, *P. multocida*, and *H. somni* from long-read sequencing based on theoretical coverage cutoffs summarized across years, sampling times and metaphylaxis groups.

	2020 (*n* = 909)						2021 (*n* = 1079)			
	Tulathromycin Metaphylaxis						Oxytetracycline Metaphylaxis		Tulathromycin Metaphylaxis	
	Theoretical Coverage Cutoff	DOF	*n*	Samples Above Cutoff	Theoretical Coverage Cutoff	DOF	*n*	Samples Above Cutoff	*n*	Samples Above Cutoff
*M.* *haemolytica*	>5.1×	Arrival processing	426	140 (33%)	>1.7×	Arrival processing	215	100 (47%)	199	98 (49%)
		13	404	138 (34%)		13	215	120 (56%)	200	74 (37%)
		36	79	56 (71%)		36	130	66 (51%)	120	72 (60%)
*P. multocida*	>1.2×	Arrival processing	426	258 (61%)	>0.26×	Arrival processing	215	92 (43%)	199	90 (45%)
		13	404	31 (7.7%)		13	215	86 (40%)	200	32 (16%)
		36	79	17 (22%)		36	130	73 (56%)	120	39 (33%)
*H. somni*	>0.09×	Arrival processing	426	50 (12%)	>0.05×	Arrival processing	215	13 (6.1%)	199	28 (14%)
		13	404	47 (12%)		13	215	34 (16%)	200	22 (11%)
		36	79	27 (34%)		36	130	94 (72%)	120	66 (55%)

DOF—days on feed.

**Table 6:** Diagnostic sensitivity and specificity of long-read metagenomic sequencing and culture for the detection of *M. haemolytica*, *P. multocida*, and *H. somni* estimated from Bayesian latent class models.

			2020 (*n* = 909)		2021 (*n* = 1079)	
Bacteria	Metric	Method	Median	95% CrI	Median	95% CrI
*M. haemolytica*	Sensitivity	Culture	0.99	0.96, 0.999	0.90	0.81, 0.996
		Sequencing ^1^	0.71	0.65, 0.78	0.91	0.87, 0.96
	Specificity	Culture	0.97	0.91, 0.999	0.99	0.95, 0.999
		Sequencing ^1^	0.92	0.89, 0.95	0.90	0.83, 0.996
*P. multocida*	Sensitivity	Culture	0.86	0.81, 0.90	0.77	0.70, 0.84
		Sequencing ^1^	0.96	0.92, 0.999	0.89	0.84, 0.94
	Specificity	Culture	0.94	0.91, 0.96	0.99	0.96, 0.999
		Sequencing ^1^	0.98	0.96, 0.999	0.97	0.93, 0.999
*H. somni*	Sensitivity	Culture	0.84	0.65, 0.999	0.79	0.73, 0.86
		Sequencing ^1^	0.52	0.36, 0.67	0.86	0.81, 0.91
	Specificity	Culture	0.97	0.95, 0.99	0.99	0.98, 0.999
		Sequencing ^1^	0.90	0.88, 0.92	0.97	0.95, 0.99

CrI—credible interval; ^1^ Sequencing classified as positive or negative based on theoretical coverage: in 2020 *M. haemolytica* > 5.1×, *P multocida* > 1.2×, *H. somni* > 0.09×; in 2021 *M. haemolytica* > 1.7×, *P. multocida* > 0.26×, *H. somni* > 0.05×.

**Table 7:** Diagnostic sensitivity (Se) and specificity (Sp) estimates from Bayesian latent class models comparing classification of *M. haemolytica*, *P. multocida*, and/or *H. somni* as non-susceptible (intermediate or resistant) to various antimicrobials by antimicrobial susceptibility testing (AST) to the detection of specific antimicrobial resistance genes (ARGs) using long-read metagenomic sequencing on deep nasopharyngeal swab samples collected from feedlot calves.

			2020 (*n* = 909)		2021 (*n* = 1076)	
Model	Metric	Method	Median	95% CrI	Median	95% CrI
AST: any macrolide ARG: *msrE-mphE*	Se	AST	0.86	0.80, 0.92	n/a	
		Seq	0.61	0.55, 0.68		
	Sp	AST	0.94	0.91, 0.96	n/a	
		Seq	0.97	0.95, 0.99		
AST: GAM or TULA ^1^ ARG: *msrE-mphE*	Se	AST	0.85	0.79, 0.91	n/a	
		Seq	0.60	0.54, 0.67		
	Sp	AST	0.95	0.92, 0.97	n/a	
		Seq	0.97	0.95, 0.98		
AST: TILD or TILM ^2^ ARG: *msrE-mphE*	Se	AST	0.58	0.50, 0.66	n/a	
		Seq	0.62	0.53, 0.70		
	Sp	AST	0.95	0.93, 0.97	n/a	
		Seq	0.97	0.95, 0.99		
AST: any macrolide ARG: *EstT*	Se	AST	0.67	0.55, 0.78	0.69	0.54, 0.84
		Seq	0.13	0.09, 0.17	0.86	0.72, 0.999
	Sp	AST	0.96	0.93, 0.999	0.97	0.95, 0.979
		Seq	0.98	0.96, 0.99	0.99	0.978, 0.998
AST: GAM or TULA ^1^ ARG: *EstT*	Se	AST	0.65	0.54, 0.77	0.38	0.26, 0.509
		Seq	0.12	0.09, 0.16	0.69	0.514, 0.85
	Sp	AST	0.96	0.93, 0.99	0.995	0.99, 0.999
		Seq	0.98	0.96, 0.99	0.99	0.99, 0.999
AST: TILD or TILM ^2^ ARG: *EstT*	Se	AST	0.52	0.39, 0.67	0.69	0.54, 0.83
		Seq	0.15	0.10, 0.20	0.92	0.79, 0.999
	Sp	AST	0.97	0.95, 0.999	0.97	0.96, 0.983
		Seq	0.98	0.97, 0.998	0.99	0.978, 0.998
AST: any macrolide ARG: *msrE-mphE* and/or *EstT*	Se	AST	0.84	0.77, 0.90	0.69	0.54, 0.83
		Seq	0.66	0.59, 0.73	0.86	0.72, 0.998
	Sp	AST	0.94	0.91, 0.96	0.97	0.95, 0.979
		Seq	0.95	0.93, 0.97	0.99	0.978, 0.998
AST: TET ARG: *tet(H)*	Se	AST	0.50	0.23, 0.85	0.59	0.50, 0.68
		Seq	0.85	0.67, 0.999	0.74	0.66, 0.83
	Sp	AST	0.99	0.98, 0.999	0.995	0.99, 0.999
		Seq	0.91	0.87, 0.97	0.99	0.97, 0.999

CrI—credible interval; AST—antimicrobial susceptibility testing; ARG—antimicrobial resistance gene; seq—long-read metagenomic sequencing; Se—sensitivity; Sp—specificity; GAM—gamithromycin; TULA—tulathromycin; TILD—tildipirosin; TILM—tilmicosin; TET—tetracycline; n/a—models not reported due to low number of samples in which *msrE* or *mphE* detected in 2021. ^1^ 15-membered ring macrolides. ^2^ 16-membered ring macrolides.

**Table 8:** Pairwise comparisons from repeated measures, multilevel logistic regression models for the likelihood of a calf within a pen having detected reads for *M. haemolytica*, *P. multocida*, or *H. somni* across time points (arrival processing prior to metaphylaxis administration (1 DOF), 13 DOF, and 36 DOF) for each year and metaphylaxis option. Results are reported as odds ratios and 95% confidence intervals (CIs), accounting for clustering at the pen level (*n* = 909 for 2020; *n* = 1076 for 2021).

Bacteria	Year/ Metaphylaxis	DOF Comparison	Odds Ratio	95% CI Lower	95% CI Upper	*p*-Value
*M. haemolytica*	2020/Tulathromycin	13 DOF vs. 1 DOF	1.1	0.80	1.4	0.65
		36 DOF vs. 1 DOF	5.5	3.2	9.4	**≤0.001**
		36 DOF vs. 13 DOF	5.1	3.0	8.8	**≤0.001**
*P. multocida*	2020/Tulathromycin	13 DOF vs. 1 DOF	0.03	0.015	0.06	**≤0.001**
		36 DOF vs. 1 DOF	0.12	0.06	0.25	**≤0.001**
		36 DOF vs. 13 DOF	4.1	1.9	8.6	**≤0.001**
*H. somni*	2020/Tulathromycin	13 DOF vs. 1 DOF	0.98	0.64	1.5	0.94
		36 DOF vs. 1 DOF	3.9	2.3	6.9	**≤0.001**
		36 DOF vs. 13 DOF	4.0	2.3	7.0	**≤0.001**
*M. haemolytica*	2021/Tulathromycin	13 DOF vs. 1 DOF	0.59	0.39	0.89	**0.012**
		36 DOF vs. 1 DOF	1.6	0.99	2.6	0.055
		36 DOF vs. 13 DOF	2.7	1.7	4.5	**≤0.001**
*P. multocida*	2021/Tulathromycin	13 DOF vs. 1 DOF	0.15	0.08	0.28	**≤0.001**
		36 DOF vs. 1 DOF	0.53	0.30	0.92	**0.025**
		36 DOF vs. 13 DOF	3.5	1.8	6.6	**≤0.001**
*H. somni*	2021/Tulathromycin	13 DOF vs. 1 DOF	0.75	0.41	1.4	0.36
		36 DOF vs. 1 DOF	8.4	4.8	15	**≤0.001**
		36 DOF vs. 13 DOF	11	6.2	20	**≤0.001**
*M. haemolytica*	2021/Oxytetracycline	13 DOF vs. 1 DOF	1.5	1.002	2.2	**0.049**
		36 DOF vs. 1 DOF	1.04	0.6	1.7	0.88
		36 DOF vs. 13 DOF	0.7	0.4	1.1	0.14
*P. multocida*	2021/Oxytetracycline	13 DOF vs. 1 DOF	0.9	0.5	1.3	0.49
		36 DOF vs. 1 DOF	2.2	1.2	3.8	**0.006**
		36 DOF vs. 13 DOF	2.6	1.4	4.5	**0.001**
*H. somni*	2021/Oxytetracycline	13 DOF vs. 1 DOF	2.9	1.5	5.7	**0.002**
		36 DOF vs. 1 DOF	46	22	94	**≤0.001**
		36 DOF vs. 13 DOF	16	8.8	28	**≤0.001**

DOF—days on feed, **bolded values** indicate statistically significant differences (*p* < 0.05).

**Table 9:** Pairwise comparisons from the repeated measures, multilevel logistic regression models for the likelihood of a calf within a pen having antimicrobial resistance genes (ARG) *msrE-mphE*, *EstT*, or *tet(H)* within *M. haemolytica*, *P. multocida*, and/or *H. somni* reads across time points (arrival processing prior to metaphylaxis administration (1 DOF), 13 DOF, and 36 DOF) for each year and metaphylaxis option. Results are reported as odds ratios and 95% confidence intervals (CIs), accounting for clustering at the pen level (*n* = 909 for 2020; *n* = 1076 for 2021).

ARG	Year/Metaphylaxis	DOF Comparison	Odds Ratio	95% CI Lower	95% CI Upper	*p*-Value
*mphE-msrE*	2020/ Tulathromycin	13 DOF vs. 1 DOF	20	11	36	**≤0.001**
		36 DOF vs. 1 DOF	42	20	88	**≤0.001**
		36 DOF vs. 13 DOF	2.1	1.3	3.6	**0.004**
*EstT*	2020/ Tulathromycin	13 DOF vs. 1 DOF	5.7	2.5	13	**≤0.001**
		36 DOF vs. 1 DOF	11	3.7	33	**≤0.001**
		36 DOF vs. 13 DOF	1.9	0.85	4.4	0.12
*tet(H)*	2020/ Tulathromycin	13 DOF vs. 1 DOF	1.1	0.71	1.6	0.78
		36 DOF vs. 1 DOF	2.5	1.4	4.6	**0.002**
		36 DOF vs. 13 DOF	2.4	1.3	4.3	**0.004**
*mphE-msrE*	2021/ Tulathromycin ^1^	13 DOF vs. 1 DOF	0.995	0.03	∞	0.99
		36 DOF vs. 1 DOF	6.5	0.69	∞	0.11
		36 DOF vs. 13 DOF	5.1	0.40	269	0.30
*EstT*	2021/ Tulathromycin ^1^	13 DOF vs. 1 DOF	50	8.7	∞	**≤0.001**
		36 DOF vs. 1 DOF	93	16	∞	**≤0.001**
		36 DOF vs. 13 DOF	1.9	1.03	3.5	**0.04**
*tet(H)*	2021/ Tulathromycin	13 DOF vs. 1 DOF	5.0	2.3	11	**≤0.001**
		36 DOF vs. 1 DOF	11	4.7	25	**≤0.001**
		36 DOF vs. 13 DOF	2.2	1.2	3.9	**0.008**
*mphE-msrE*	2021/ Oxytetracycline	13 DOF vs. 1 DOF	n/a			
		36 DOF vs. 1 DOF	n/a			
		36 DOF vs. 13 DOF	n/a			
*EstT*	2021/ Oxytetracycline	13 DOF vs. 1 DOF	5.5	0.94	32	0.058
		36 DOF vs. 1 DOF	2.4	0.27	21	0.43
		36 DOF vs. 13 DOF	0.44	0.07	2.6	0.37
*tet(H)*	2021/ Oxytetracycline	13 DOF vs. 1 DOF	20	6.8	57	**≤0.001**
		36 DOF vs. 1 DOF	32	9.9	100	**≤0.001**
		36 DOF vs. 13 DOF	1.6	0.88	2.9	0.12

DOF—days on feed, **bolded values** indicate statistically significant differences (*p* < 0.05). ^1^ Low number of positive samples. Exact logistic regression used. Median unbiased estimate reported. n/a—Models unable to run due to zero samples with *msrE* or *mphE* detected.

## Data Availability

The genomic data used in this study have been deposited in the Sequence Reach Archive (SRA) within Submission SUB15731984. Custom scripts can be accessed at: https://github.com/coadunate/ASSETS_2 (accessed on 26 October 2025).

## Supplementary Materials

The following supporting information can be downloaded at: https://www.mdpi.com/article/10.3390/antibiotics14111114/s1, Table S1. Summary of differences in sample handling, preparation and sequencing by sampling year. Table S2. Antimicrobial resistance genes (ARGs) detected in at least five samples by long-read metagenomic sequencing in reads identified as *M. haemolytica*, *P. multocida*, and *H. somni* from 2020 and 2021 (*n* = 1985 samples). Table S3. Comparison of sensitivity (Se) and specificity (Sp) estimates for BLCM models for detection of *M. haemolytica*, *P. multocida* and *H. somni* with and without covariance terms (2020 *n* = 909; 2021 *n* = 1079). Table S4. Sensitivity (Se) and specificity (Sp) estimates from Bayesian latent class models comparing classification of *M. haemolytica*, *P. multocida* and/or *H. somni* as susceptible or non-susceptible by antimicrobial susceptibility testing (AST) to the detection of *msrE-mphE* or *EstT* using long-read metagenomic sequencing. Models in which intermediate AST results based on CLSI breakpoints were considered susceptible were compared to models in which intermediate AST results were considered resistant. Table S5. Comparison of sensitivity (Se) and specificity (Sp) estimates for BLCM models for detection of *M. haemolytica*, *P. multocida* and *H. somni* for models considering only the time 2 (13 DOF) and 3 (36 DOF) populations and models including all populations (time 1 (arrival processing), 2 (13 DOF) and 3 (36 DOF)) to evaluate the assumption that test performance was constant across populations. Table S6. Comparison of sensitivity (Se) and specificity (Sp) estimates from Bayesian latent class models comparing classification of *M. haemolytica*, *P. multocida* and/or *H. somni* as susceptible or non-susceptible (intermediate included with resistant isolates identified as non-susceptible) by antimicrobial susceptibility testing to the detection of specific antimicrobial resistance genes using long-read metagenomic sequencing for models considering only the time 2 (13 DOF) and 3 (36 DOF) populations and models including all populations (time 1 (on arrival), 2 (13 DOF) and 3 (36 DOF)) to evaluate the assumption that test performance was constant across populations. Table S7. Sensitivity (Se) and specificity (Sp) estimates for detection of macrolide-resistant *M. haemolytica* from deep nasopharyngeal swab samples collected from feedlot calves. Estimates compare antimicrobial susceptibility testing (AST) results for tulathromycin and tilmicosin resistance with metagenomic sequencing detection of ARGs *mphE-msrE* and *EstT*, using two-test, three-population Bayesian latent class models without informative priors (*n* = 909). Table S8. Positive and negative predictive values for detection of bacteria from long-read metagenomics estimated based on sensitivity and specificity from Bayesian latent class models. Table S9. Positive and negative predictive values for detection of ARGs associated with respiratory bacteria by long-read metagenomics based on sensitivity and specificity from Bayesian latent class models.

## Abbreviations

The following abbreviations are used in this manuscript:

AMR	Antimicrobial Resistance
AMU	Antimicrobial Use
ARG	Antimicrobial Resistance Gene
AST	Antimicrobial Susceptibility Testing
BHI	Brain Heart Infusion Broth
BLCM	Bayesian Latent Class Model
BRD	Bovine Respiratory Disease
BW	Body Weight
CARD	Comprehensive Antimicrobial Resistance Database
CCAC	Canadian Council of Animal Care
CLSI	Clinical and Laboratory Standards Institute
CrI	Credible Intervals
C/S	Culture and Antimicrobial Susceptibility Testing
DHPR	Dihydrofolate Reductase
DNPS	Deep Nasopharyngeal Swab
DOF	Days on Feed
MIC	Minimum Inhibitory Concentration
NPV	Negative Predictive Value
OIE	World Organization for Animal Health
ONT	Oxford Nanopore Technologies
PBS	Phosphate-Buffered Saline
PCR	Polymerase Chain Reaction
PPV	Positive Predictive Value
Se	Sensitivity
Sp	Specificity
