# Supplementary material for: Evaluating the Diagnostic Performance of Long-Read Metagenomic Sequencing Compared to Culture and Antimicrobial Susceptibility Testing for Detection of Bovine Respiratory Bacteria and Indicators of Antimicrobial Resistance
Source: Antibiotics (Basel). 2025 Nov 5;14(11):1114. doi: 10.3390/antibiotics14111114 (PMC12649616; doi:10.3390/antibiotics14111114)
Supplement: Supplementary file 1 [file antibiotics-14-01114-s001.zip › antibiotics-3934705-supplementary.pdf]

Supplementary Materials

Evaluating the diagnostic performance of long-read metagenomic sequencing compared to culture and antimicrobial susceptibility testing for detection of bovine respiratory bacteria and indicators of antimicrobial resistance

Abi Younes, J.N.[1], McLeod, L.[1], Otto, S.J.G.[2,3], Chai, Z.[1], Lacoste, S.[1], McCarthy, E.L.[4], Links, M.G.[4], Herman, E.K.[5], Stothard, P.[6], Gow, S.P.[7], Campbell, J.R.[1], Waldner, C.L.[1,*]

[1] Department of Large Animal Clinical Sciences, Western College of Veterinary Medicine, University of Saskatchewan, Saskatoon, SK, Canada
[2] HEAT-AMR (Human-Environment-Animal Transdisciplinary AMR) Research Group, School of Public Health, University of Alberta, Edmonton, AB, Canada
[3] Centre for Healthy Communities, School of Public Health, University of Alberta, Edmonton, AB, Canada
[4] Department of Animal and Poultry Science, College of Agriculture and Bioresources, University of Saskatchewan, Saskatoon, SK, Canada
[5] [5]Department of Biochemistry and Medical Genetics, Max Rady College of Medicine, University of Manitoba, Winnipeg, MB, Canada
[6] Department of Agricultural, Food, and Nutritional Science, Faculty of Agricultural, Life, and Environmental Sciences, University of Alberta, Edmonton, AB, Canada
[7] Canadian Integrated Program for Antimicrobial Resistance Surveillance, Public Health Agency of Canada, Saskatoon, SK, Canada
* Correspondence: cheryl.waldner@usask.ca

Contents:

- **Differences in sample handling, preparation and sequencing across years**
- **Antimicrobial resistance gene detail**
- **Sensitivity analysis for inclusion of covariance terms in BLCM for detection of bacteria by culture and metagenomic long-read sequencing**
- **Sensitivity analysis for consistency of test performance across populations**
- **Sensitivity analysis to compare test performance in *M. haemolytica* for specific antimicrobial and gene combinations**
- **Calculation of positive and negative predictive values based on the results of the Bayesian latent class models**

Differences in sample handling, preparation and sequencing across years

Table S1. Summary of differences in sample handling, preparation and sequencing by sampling year.

Sampling Year	Source	Handling	Enrichment, host depletion, extraction	Library preparation and sequencing
2021	USask feedlot	Transported directly to lab (within 2 hours)	10-hour enrichment<br>No host depletion<br>Extraction: 1.5 mL culture media<br>Completed by ASSETS lab team	ONT Ligation sequencing gDNA - native barcoding (SQK-LSK109 with EXP-NBD196)<br>80-200 ng per library<br>R9.4.1 flow cell<br>72 hours sequencing time<br>32 samples/library
2020<br>(Metagenomic sequencing completed after 2021 samples)	USask feedlot	Transported directly to lab (within 2 hours)<br>Swab heads frozen (-80°C)	14-hour enrichment<br>Extraction: 1.5 mL culture media<br>Completed by ASSETS lab team	ONT Ligation sequencing gDNA - native barcoding (SQK-LSK109 with EXP-NBD196)<br>70-150 per library<br>R9.4.1 flow cell<br>48 hours sequencing time<br>32 samples/library

Antimicrobial resistance gene detail

Table S2. Antimicrobial resistance genes (ARGs) detected in at least five samples by long-read metagenomic sequencing in reads identified as *M. haemolytica*, *P. multocida*, and *H. somni* from 2020 and 2021 (*n*=1,985 samples).

Gene	Total (*n*=1985)	2020 (*n*=909)	2021 (*n*=1076)
sul2	327 (16.5%)	234 (25.7%)	93 (8.6%)
tet(H)	313 (15.8%)	138 (15.2%)	175 (16.3%)
mphE	181 (9.1%)	178 (19.6%)	3 (0.3%)
msrE	177 (8.9%)	174 (19.1%)	3 (0.3%)
APH(3'')-Ib	165 (8.3%)	85 (9.4%)	80 (7.4%)
APH(3')-Ia	160 (8.1%)	84 (9.2%)	76 (7.1%)
APH(6)-Id	157 (7.9%)	81 (8.9%)	76 (7.1%)
EstT	131 (6.6%)	60 (6.6%)	71 (6.6%)
aadA14	33 (1.7%)	12 (1.3%)	21 (2.0%)
emrR	30 (1.5%)	29 (3.2%)	1 (0.1%)
CRP	29 (1.5%)	26 (2.9%)	3 (0.3%)
aadA31	23 (1.2%)	8 (0.9%)	15 (1.4%)
H-NS	23 (1.2%)	22 (2.4%)	1 (0.1%)
bacA	21 (1.1%)	19 (2.1%)	2 (0.2%)
AcrS	19 (1.0%)	19 (2.1%)	0
cpxA	19 (1.0%)	19 (2.1%)	0
floR	18 (0.9%)	18 (2.0%)	0
kdpE	18 (0.9%)	18 (2.0%)	0
mdtN	18 (0.9%)	18 (2.0%)	0
PmrF	18 (0.9%)	16 (1.8%)	2 (0.2%)
marA	16 (0.8%)	15 (1.7%)	1 (0.1%)
mdtP	16 (0.8%)	16 (1.8%)	0
ugd	16 (0.8%)	15 (1.7%)	1 (0.1%)
astA	14 (0.7%)	13 (1.4%)	1 (0.1%)
gadX	14 (0.7%)	13 (1.4%)	1 (0.1%)
leuO	14 (0.7%)	11 (1.2%)	3 (0.3%)
Escherichia_coli_acrA	13 (0.7%)	12 (1.3%)	1 (0.1%)
Escherichia_coli_emrE	12 (0.6%)	11 (1.2%)	1 (0.1%)
YojI	12 (0.6%)	10 (1.1%)	2 (0.2%)
emrA	11 (0.6%)	10 (1.1%)	1 (0.1%)
emrB	11 (0.6%)	10 (1.1%)	1 (0.1%)
iss	11 (0.6%)	10 (1.1%)	1 (0.1%)
mdtE	11 (0.6%)	11 (1.2%)	0
mdtG	11 (0.6%)	10 (1.1%)	1 (0.1%)
msbA	11 (0.6%)	11 (1.2%)	0
OmpA	11 (0.6%)	10 (1.1%)	1 (0.1%)
gadW	10 (0.5%)	10 (1.1%)	0
mdtA	10 (0.5%)	10 (1.1%)	0
EC-18	9 (0.5%)	7 (0.8%)	2 (0.2%)
evgA	9 (0.5%)	9 (1.0%)	0
mdtH	9 (0.5%)	7 (0.8%)	2 (0.2%)
mdtM	9 (0.5%)	9 (1.0%)	0
AcrE	8 (0.4%)	8 (0.9%)	0

**Gene**	**Total (*n*=1985)**	**2020 (*n*=909)**	**2021 (*n*=1076)**
eptA	8 (0.4%)	8 (0.9%)	0
Erm(42)	8 (0.4%)	8 (0.9%)	0
mdtO	8 (0.4%)	8 (0.9%)	0
TolC	8 (0.4%)	8 (0.9%)	0
arsR	6 (0.3%)	6 (0.7%)	0
emrY	6 (0.3%)	5 (0.6%)	1 (0.1%)
Escherichia_coli_mdfA	6 (0.3%)	6 (0.7%)	0
acrD	5 (0.3%)	3 (0.3%)	2 (0.2%)
AcrF	5 (0.3%)	5 (0.6%)	0
arsC	5 (0.3%)	5 (0.6%)	0
asr	5 (0.3%)	5 (0.6%)	0
emrK	5 (0.3%)	5 (0.6%)	0
mdtB	5 (0.3%)	5 (0.6%)	0
tet(38)	5 (0.3%)	5 (0.6%)	0

Sensitivity analysis for inclusion of covariance terms in BLCM for detection of bacteria by culture and metagenomic long-read sequencing

Latent class modeling for diagnostic test assessment in the absence of a gold standard makes the assumptions that the tests being evaluated are conditionally independent. However, the introduction of covariance terms between sensitivity and specificity between the tests with Bayesian latent class modeling (BLCM) offers a method to account for tests that are not independent. While culture and long-read metagenomic sequencing employ fundamentally different processes so could be considered independent, the non-specific culture-based enrichment step used prior to sequencing in this study had the potential to induce dependence between the tests in this case. To test the assumption of conditional independence, models were run with and without covariance following model structures similar to those described in the main paper. The primary exception was that each of three chains was run for 100,000 iterations in each of the models described in Table S2. This improved the diagnostic metrics for these models, as the introduction of extra parameters in these models negatively impacted effective sample size in some models.

The introduction of covariance terms in the *M. haemolytica* models resulted in implausible estimates for the specificity of culture for both 2020 and 2021 (Table S3), confirmed by comparison to unpublished whole genome sequencing (WGS) completed on culture positive samples. In addition, convergence diagnostics and very wide credible intervals suggested the estimates from these models were potentially unreliable. To mitigate this issue, a relatively strong prior on the specificity of culture (beta [100, 1.5]) was introduced to the *M. haemolytica* models both with and without covariance terms. This prior was based on the unpublished WGS results and derived via https://shiny.vet.unimelb.edu.au/epi/beta.buster/, using a most likely value for culture specificity of 0.995, with 95% confidence the value was > 0.975. The models with a prior on culture specificity produced estimates with overlapping CrIs with or without covariance terms. The 95% credible intervals for the covariance terms included zero, suggesting the covariance terms were not important to the models. Excluding covariance, the estimates from the models with and without the prior were very similar and DIC varied minimally (≤ 3) across all model configurations; therefore, the most parsimonious model (without covariance terms or priors) was reported in the main manuscript.

For *P. multocida* and *H. somni* in both 2020 and 2021 (Table S3), the introduction of covariance terms had less impact on the models. The 95% credible intervals for covariance terms for both sensitivity and specificity included the zero, suggesting these terms were not important to the models. The credible intervals for sensitivity and specificity also exhibited considerable overlap in models with and without covariance terms. Given the introduction of extra terms to the model resulted in greater uncertainty in estimates (wider CrIs) and minimal change in DIC, the more parsimonious models without covariance were reported in the main manuscript.

Sensitivity analysis comparing models in which intermediate AST results were considered susceptible and models in which intermediate AST results were considered resistant

Diagnostic sensitivity (Se) and specificity (Sp) estimates were reported for BLCM comparing classification of *M. haemolytica, P. multocida* and/or *H. somni* as susceptible or non-susceptible by antimicrobial susceptibility testing (AST) to the detection of *msrE-mphE* or *EstT* using long-read metagenomic sequencing for both models in which intermediate AST results were considered susceptible and models in which intermediate AST results were considered resistant (Table S4).

In most models, the estimates for long-read metagenomic test sensitivity and specificity were similar models in which intermediate AST results were considered susceptible and models in which intermediate AST results were considered resistant. The one exception (Table S4) was the BLCM model for AST: TILD or TILM and ARG: *EstT* where the sensitivity estimates for *EstT* improved significantly when susceptible and intermediate were compared to resistant. The reductions in diagnostic Se for AST detection of AMR when intermediate was considered susceptible were expected.

Table S3. Comparison of sensitivity (Se) and specificity (Sp) estimates for Bayesian latent class models for detection of *M. haemolytica, P. multocida* and *H. somni* with and without covariance terms (2020 *n*=909; 2021 *n*=1079).

		2020				2021			
		No covariance		*With covariance*		*No covariance*		*With covariance*	
		**Median**	**95% CrI**	**Median**	**95% CrI**	**Median**	**95% CrI**	**Median**	**95% CrI**
M. haemolytica									
Se	Culture	0.99	0.96, 0.999	0.76	0.62, 0.999	0.90	0.81, 0.996	0.68	0.51, 0.96
	Sequencing[1]	0.71	0.65, 0.78	0.51	0.38, 0.68	0.91	0.87, 0.96	0.66	0.51, 0.87
Sp	Culture	0.97	0.91, 0.999	0.78	0.66, 0.98[2]	0.99	0.95, 0.999	0.72	0.62, 0.87[2]
	Sequencing[1]	0.92	0.89, 0.95	0.74	0.66, 0.90	0.90	0.83, 0.99	0.62	0.52, 0.77
Se covariance		n/a		0.11	-0.001, 0.15	n/a		0.13	-0.001, 0.19
Sp covariance		n/a		0.12	0.01, 0.16	n/a		0.16	0.09, 0.20
M. haemolytica with prior on culture Sp									
Se	Culture	0.99	0.96, 0.999	0.82	0.65, 0.999	0.90	0.81, 0.997	0.74	0.54, 0.97
	Sequencing[1]	0.70	0.65, 0.74	0.58	0.45, 0.71	0.91	0.87, 0.94	0.75	0.57, 0.92
Sp	Culture	0.99	0.97, 0.999	0.99	0.96, 0.999	0.99	0.97, 0.999	0.99	0.96, 0.999
	Sequencing[1]	0.92	0.89, 0.95	0.90	0.84, 0.94	0.90	0.83, 0.99	0.87	0.78, 0.99
Se covariance		n/a		0.09	-0.002, 0.15	n/a		0.11	-0.003, 0.19
Sp covariance		n/a		0.004	-0.002, 0.02	n/a		0.004	-0.002, 0.02
P. multocida									
Se	Culture	0.86	0.81, 0.90	0.77	0.57, 0.88	0.77	0.70, 0.84	0.59	0.42, 0.79
	Sequencing[1]	0.96	0.92, 0.999	0.87	0.63, 0.999	0.89	0.84, 0.95	0.69	0.50, 0.92
Sp	Culture	0.94	0.91, 0.96	0.92	0.87, 0.95	0.99	0.96, 0.999	0.93	0.85, 0.999
	Sequencing[1]	0.98	0.96, 0.999	0.96	0.92, 0.998	0.97	0.93, 0.999	0.91	0.82, 0.999
Se covariance		n/a		0.07	-0.004, 0.17	n/a		0.11	-0.01, 0.17
Sp covariance		n/a		0.01	-0.002, 0.05	n/a		0.04	-0.001, 0.10
H. somni									
Se	Culture	0.84	0.65, 0.999	0.77	0.49, 0.999	0.79	0.73, 0.86	0.72	0.59, 0.84
	Sequencing[1]	0.52	0.36, 0.67	0.45	0.27, 0.64	0.86	0.81, 0.92	0.78	0.65, 0.90
Sp	Culture	0.97	0.95, 0.99	0.97	0.94, 0.996	0.99	0.98, 0.999	0.98	0.93, 0.999
	Sequencing[1]	0.90	0.88, 0.92	0.90	0.87, 0.92	0.97	0.95, 0.99	0.95	0.90, 0.99
Se covariance		n/a		0.04	-0.04, 0.12	n/a		0.05	-0.02, 0.12
Sp covariance		n/a		0.002	-0.004, 0.01	n/a		0.01	-0.0004, 0.05

[1]Sequencing classified as positive or negative based on theoretical coverage: 2020 *M. haemolytica* > 5.1, *P multocida* > 1.2, *H. somni* > 0.09; 2021 *M. haemolytica* > 1.7, *P multocida* > 0.26, *H. somni* > 0.05. [2] Estimated median < 80% and in 2021 95%CrI did not contain 90% and as such considered implausible based on unpublished WGS data.

Table S4. Sensitivity (Se) and specificity (Sp) estimates from Bayesian latent class models comparing classification of *M. haemolytica, P. multocida* and/or *H. somni* as susceptible or non-susceptible by antimicrobial susceptibility testing (AST) to the detection of *msrE-mphE* or *EstT* using long-read metagenomic sequencing. Models in which intermediate AST results based on CLSI breakpoints were considered susceptible were compared to models in which intermediate AST results were considered resistant.

			**2020** (*n*=909)				**2021** (*n*=1076)			
			**Intermediate as susceptible**		**Intermediate as resistant**		**Intermediate as susceptible**		**Intermediate as resistant**	
**Model**	**Metric**	**Method**	**Median**	**95% CrI**	**Median**	**95% CrI**	**Median**	**95% CrI**	**Median**	**95% CrI**
AST: any macrolide ARG: *msrE-mphE*	Se	AST	0.84	0.77, 0.90	0.86	0.80, 0.92	n/a		n/a	
		Seq	0.62	0.56, 0.68	0.61	0.55, 0.68				
	Sp	AST	0.99	0.97, 0.997	0.94	0.91, 0.96	n/a		n/a	
		Seq	0.97	0.95, 0.98	0.97	0.95, 0.99				
AST: any macrolide ARG: *EstT*	Se	AST	0.55	0.47, 0.66	0.67	0.55, 0.78	**0.39**	**0.27, 0.52**	**0.69**	**0.54, 0.84**
		Seq	0.12	0.08, 0.15	0.13	0.09, 0.17	0.79	0.63, 0.92	0.86	0.72, 0.999
	Sp	AST	0.99	0.98, 0.999	0.96	0.93, 0.999	0.999	0.996, 0.999	0.97	0.95, 0.98
		Seq	0.98	0.96, 0.99	0.98	0.96, 0.99	0.99	0.98, 0.999	0.99	0.98, 0.998
AST: any macrolide ARG: *msrE-mphE and/or EstT*	Se	AST	0.80	0.74, 0.87	0.84	0.77, 0.90	**0.39**	**0.27, 0.52**	**0.69**	**0.54, 0.83**
		Seq	0.66	0.60, 0.72	0.66	0.59, 0.73	0.79	0.64, 0.92	0.86	0.72, 0.998
	Sp	AST	0.99	0.97, 0.996	0.94	0.91, 0.96	0.999	0.996, 0.999	0.97	0.95, 0.98
		Seq	0.95	0.93, 0.97	0.95	0.93, 0.97	0.99	0.98, 0.999	0.99	0.98, 0.998
AST: GAM or TULA ARG: *msrE-mphE*	Se	AST	0.83	0.77, 0.90	0.85	0.79, 0.91	n/a		n/a	
		Seq	0.63	0.57, 0.70	0.60	0.54, 0.67				
	Sp	AST	0.99	0.97, 0.996	0.95	0.92, 0.97	n/a		n/a	
		Seq	0.97	0.95, 0.98	0.97	0.95, 0.98				
AST: GAM or TULA ARG: *EstT*	Se	AST	0.53	0.45, 0.63	0.65	0.54, 0.77	0.24	0.14, 0.36	0.38	0.26, 0.51
		Seq	0.12	0.09, 0.15	0.12	0.09, 0.16	0.73	0.52, 0.91	0.69	0.51, 0.85
	Sp	AST	0.99	0.98, 0.999	0.96	0.93, 0.99	0.999	0.996, 0.999	0.995	0.99, 0.999
		Seq	0.98	0.96, 0.99	0.98	0.96, 0.99	0.99	0.99, 0.999	0.99	0.99, 0.999
AST: TILD or TILM ARG: *msrE-mphE*	Se	**AST**	**0.07**	**0.04, 0.10**	**0.58**	**0.50, 0.66**	n/a		n/a	
		Seq	0.54	0.42, 0.67	0.62	0.53, 0.70				
	Sp	AST	0.996	0.99, 0.999	0.95	0.93, 0.97	n/a		n/a	
		Seq	0.98	0.96, 0.999	0.97	0.95, 0.99				
AST: TILD or TILM ARG: *EstT*	Se	**AST**	**0.23**	**0.10, 0.38**	**0.52**	**0.39, 0.67**	**0.39**	**0.27, 0.52**	**0.69**	**0.54, 0.83**
		**Seq**	**0.46**	**0.24, 0.70**	**0.15**	**0.10, 0.20**	0.79	0.63, 0.93	0.92	0.79, 0.999
	Sp	AST	0.997	0.99, 0.999	0.97	0.95, 0.999	0.999	0.996, 0.999	0.97	0.96, 0.98
		Seq	0.98	0.96, 0.998	0.98	0.97, 0.998	0.99	0.98, 0.999	0.99	0.98, 0.998
AST: TET ARG: *tet(H)*	Se	AST	0.20	0.06, 0.49	0.50	0.23, 0.85	0.50	0.40, 0.62	0.59	0.50, 0.68
		Seq	0.78	0.54, 0.999	0.85	0.67, 0.999	0.71	0.62, 0.79	0.74	0.66, 0.83
	Sp	AST	0.99	0.99, 0.999	0.99	0.98, 0.999	0.998	0.99, 0.999	0.995	0.99, 0.999
		Seq	0.92	0.87, 0.99	0.91	0.87, 0.97	0.98	0.95, 0.999	0.99	0.97, 0.999

CrI – credible interval; AST – antimicrobial susceptibility testing; ARG – antimicrobial resistance gene; seq – long-read metagenomic sequencing; Se – sensitivity; Sp – specificity; GAM – gamithromycin; TULA – tulathromycin; TILD – tildipirosin; TILM – tilmicosin; TET – tetracycline; n/a – models not reported due to low number of samples in which *msrE* or *mphE* detected in 2021.

Sensitivity analysis for consistency of test performance across populations

Additional models were constructed to assess the sensitivity of the models for bacteria (Table S5) and AMR/ARG detection (Table S6) to the assumption that test performance was consistent among populations. Because the prevalence of the BRD bacteria and AMR determinants was expected to be considerably different in the on-arrival (1 day on feed [DOF]) samples compared to the other time points, models were run excluding this population and estimates were compared to the full models. For the 2020 models, the loss of information resulting from dropping these data resulted in implausible estimates for detection of *M. haemolytica* and phenotypic macrolide AMR by culture and AST, confirmed by unpublished whole genome sequencing (WGS) completed on culture positive samples. To mitigate the loss of data in these models, a relatively strong prior on the specificity of culture or AST for macrolide resistance (beta [100, 1.5]) was introduced. The prior was based on the unpublished WGS results and derived via https://shiny.vet.unimelb.edu.au/epi/beta.buster/, using a most likely value for culture specificity of 0.995, with 95% confidence the value was greater than 0.975.

In all models, the estimates for test sensitivity and specificity were similar with and without the inclusion of the on-arrival population (1 DOF), supporting the assumption that test performance was consistent across populations.

Table S5. Comparison of sensitivity (Se) and specificity (Sp) estimates for Bayesian latent class models models for detection of *M. haemolytica, P. multocida* and *H. somni* for models considering only the time 2 (13 DOF) and 3 (36 DOF) populations and models including all populations (time 1 (arrival processing), 2 (13 DOF) and 3 (36 DOF)) to evaluate the assumption that test performance was constant across populations.

			2020				2021			
			Time 2 and 3 (*n*=483)		Time 1, 2 and 3 (*n*=909)		Time 2 and 3 (*n*=665)		Time 1, 2 and 3 (*n*=1079)	
**Model**	**Metric**	**Method**	**Median**	**95% CrI**	**Median**	**95% CrI**	**Median**	**95% CrI**	**Median**	**95% CrI**
M. haemolytica	Se	Culture	0.97	0.94, 0.999	0.99	0.96, 0.999	0.85	0.76, 0.98	0.90	0.81, 0.996
		Seq[1]	0.66	0.60, 0.72	0.71	0.65, 0.78	0.92	0.88, 0.97	0.91	0.87, 0.96
	Sp	Culture	0.98	0.95, 0.999	0.97	0.91, 0.999	0.99	0.95, 0.999	0.99	0.95, 0.999
		Seq[1]	0.98	0.95, 0.999	0.92	0.89, 0.95	0.90	0.81, 0.998	0.90	0.83, 0.996
P. multocida	Se	Culture	0.81	0.66, 0.997	0.86	0.81, 0.90	0.70	0.63, 0.78	0.77	0.70, 0.84
		Seq[1]	0.83	0.58, 0.999	0.96	0.92, 0.999	0.93	0.87, 0.99	0.89	0.84, 0.94
	Sp	Culture	0.95	0.92, 0.99	0.94	0.91, 0.96	0.98	0.95, 0.999	0.99	0.96, 0.999
		Seq[1]	0.99	0.96, 0.999	0.98	0.96, 0.999	0.98	0.94, 0.999	0.97	0.93, 0.999
H. somni	Se	Culture	0.86	0.66, 0.999	0.84	0.65, 0.999	0.79	0.73, 0.86	0.79	0.73, 0.86
		Seq[1]	0.53	0.37, 0.70	0.52	0.36, 0.67	0.87	0.81, 0.92	0.86	0.81, 0.91
	Sp	Culture	0.97	0.95, 0.996	0.97	0.95, 0.99	0.99	0.97, 0.999	0.99	0.98, 0.999
		Seq[1]	0.89	0.86, 0.92	0.90	0.88, 0.92	0.97	0.95, 0.998	0.97	0.95, 0.99

CrI – credible interval; Seq – long-read metagenomic sequencing; Se – sensitivity; Sp – specificity.

[1]Sequencing classified as positive or negative based on theoretical coverage: in 2020 *M. haemolytica* > 5.1X, *P multocida* > 1.2X, *H. somni* > 0.09X; in 2021 *M. haemolytica* > 1.7X, *P multocida* > 0.26X, *H. somni* > 0.05X

Table S6. Comparison of sensitivity (Se) and specificity (Sp) estimates from Bayesian latent class models comparing classification of *M. haemolytica, P. multocida* and/or *H. somni* as susceptible or non-susceptible (intermediate included with resistant isolates identified as non-susceptible) by antimicrobial susceptibility testing to the detection of specific antimicrobial resistance genes using long-read metagenomic sequencing for models considering only the time 2 (13 DOF) and 3 (36 DOF) populations and models including all populations (time 1 (on arrival), 2 (13 DOF) and 3 (36 DOF)) to evaluate the assumption that test performance was constant across populations.

			**2020**				**2021**			
			**Time 2 and 3** (*n*=483)		**Time 1, 2 and 3** (*n*=909)		**Time 2 and 3** (*n*=664)		**Time 1, 2 and 3** (*n*=1,076)	
**Model**	**Metric**	**Method**	**Median**	**95% CrI**	**Median**	**95% CrI**	**Median**	**95% CrI**	**Median**	**95% CrI**
AST: Any macrolide<br>ARG: *msrE-mphE*	Se	AST	0.89	0.81, 0.996	0.86	0.80, 0.92	n/a		n/a	
		seq	0.59	0.53, 0.65	0.61	0.55, 0.68				
	Sp	AST	0.99	0.96, 0.999	0.94	0.91, 0.96	n/a		n/a	
		seq	0.94	0.87, 0.999	0.97	0.95, 0.99				
AST: Any macrolide<br>ARG: *EstT*	Se	AST	0.78	0.63, 0.999	0.67	0.55, 0.78	0.78	0.62, 0.95	0.69	0.54, 0.84
		seq	0.12	0.09, 0.16	0.13	0.09, 0.17	0.85	0.71, 0.999	0.86	0.72, 0.999
	Sp	AST	0.99	0.96, 0.999	0.96	0.93, 0.999	0.97	0.95, 0.99	0.97	0.95, 0.98
		seq	0.92	0.88, 0.98	0.98	0.96, 0.99	0.97	0.95, 0.99	0.99	0.98, 0.998
AST: Any macrolide<br>ARG: *msrE-mphE and/or EstT*	Se	AST	0.86	0.77, 0.98	0.84	0.77, 0.90	0.78	0.61, 0.95	0.69	0.54, 0.83
		seq	0.64	0.58, 0.70	0.66	0.59, 0.73	0.85	0.71, 0.995	0.86	0.72, 0.998
	Sp	AST	0.99	0.96, 0.999	0.94	0.91, 0.96	0.97	0.95, 0.99	0.97	0.95, 0.98
		seq	0.93	0.83, 0.999	0.95	0.93, 0.97	0.97	0.95, 0.99	0.99	0.98, 0.998
AST: GAM or TULA<br>ARG: *msrE-mphE*	Se	AST	0.88	0.79, 0.99	0.85	0.79, 0.91	n/a		n/a	
		seq	0.59	0.52, 0.65	0.60	0.54, 0.67				
	Sp	AST	0.99	0.96, 0.999	0.95	0.92, 0.97	n/a		n/a	
		seq	0.94	0.87, 0.999	0.97	0.95, 0.98				
AST: GAM or TULA<br>ARG: *EstT*	Se	AST	0.75	0.60, 0.99	0.65	0.54, 0.77	0.42	0.29, 0.57	0.38	0.26, 0.51
		seq	0.12	0.09 0.16	0.12	0.09, 0.16	0.71	0.53, 0.87	0.69	0.51, 0.85
	Sp	AST	0.99	0.96, 0.999	0.96	0.93, 0.99	0.99	0.98, 0.999	0.995	0.99, 0.999
		seq	0.92	0.87, 0.98	0.98	0.96, 0.99	0.98	0.97, 0.999	0.99	0.99, 0.999
AST: TILM or TILD<br>ARG: *msrE-mphE*	Se	AST	0.66	0.51, 0.91	0.58	0.50, 0.66	n/a		n/a	
		seq	0.59	0.52, 0.67	0.62	0.53, 0.70				
	Sp	AST	0.99	0.96 0.999	0.95	0.93, 0.97	n/a		n/a	
		seq	0.89	0.77, 0.999	0.97	0.95, 0.99				
AST: TILM or TILD<br>ARG: *EstT*	Se	AST	0.64	0.43, 0.97	0.52	0.39, 0.67	0.78	0.61, 0.95	0.69	0.54, 0.83
		seq	0.14	0.10, 0.20	0.15	0.10, 0.20	0.91	0.79, 0.999	0.92	0.79, 0.999
	Sp	AST	0.99	0.96, 0.999	0.97	0.95, 0.999	0.98	0.96, 0.99	0.97	0.96, 0.98
		seq	0.94	0.89, 0.996	0.98	0.97, 0.998	0.97	0.95, 0.99	0.99	0.98, 0.998
AST: TET<br>ARG *tet(H)*	Se	AST	0.56	0.25, 0.93	0.50	0.23, 0.85	0.74	0.56, 0.97	0.59	0.50, 0.68
		seq	0.87	0.65, 0.999	0.85	0.67, 0.999	0.77	0.66, 0.90	0.74	0.66, 0.83
	Sp	AST	0.98	0.97, 0.999	0.99	0.98, 0.999	0.99	0.95, 0.999	0.995	0.99, 0.999
		seq	0.90	0.85, 0.97	0.91	0.87, 0.97	0.92	0.86, 0.99	0.99	0.97, 0.999

CrI – credible interval; AST – antimicrobial susceptibility testing; ARG – antimicrobial resistance gene; seq – long-read metagenomic sequencing; Se – sensitivity; Sp – specificity; GAM – gamithromycin; TULA – tulathromycin; TILD – tildipirosin; TILM – tilmicosin; TET – tetracycline; n/a – models not reported due to low number of samples in which *msrE* or *mphE* detected in 2021.

Sensitivity analysis to compare test performance in *M. haemolytica* for specific antimicrobial and gene combinations

Further BLCMs were developed to compare the sensitivity and specificity for detection of *M. haemolytica* with phenotypic resistance to specific macrolides to that of metagenomic sequencing's detection ARGs identified as potential determinants of specific macrolide resistance in *M. haemolytica* [2]. Analysis was limited to the 2020 data due to the low prevalence of *msrE* and *mphE* genes in 2021.

In these models, the latent class was defined as the potential for antimicrobial resistance in *M. haemolytica* to macrolides, which could influence clinical treatment outcomes and the transmission of resistant organisms or genes to other animals. The model comparisons included phenotypic resistance to tulathromycin and the presence of *msrE-mphE* genes and tilmicosin resistance and the *EstT* gene.

For AST, a positive test result was defined as the isolation of *M. haemolytica* from DNP swabs collected from individual calves, followed by classification of the isolate as resistant to tulathromycin or tilmicosin. For these models, intermediate results on AST were considered as susceptible facilitating comparison to the results in table S4 where resistance and detection of genes was considered across multiple organisms.

For long read metagenomic sequencing, a positive result was defined as a sample where there was detection of either of the ARGs *mphE or msrE*, or *EstT* within at least one *M. haemolytica* read. As in the primary analysis, the detection of *mphE* and *msrE* was considered in combination and defined as "either" or "both," since these genes are typically arranged in tandem and co-expressed from the same promoter [3].

Model structure and implementation was as described for the primary analysis; models were developed for 2020 only due to low numbers of macrolide ARGs detected in 2021.

Results of models comparing *M. haemolytica* resistance determined by AST to the detection of specific ARGs on *M. haemolytica* reads (Table S7) were similar to those from models comparing grouped phenotypic AST results and detection of *msrE-mphE* but were different for *EstT* in any of *M. haemolytica, P. multocida* or *H. somni* (Table S4). The Se estimates were significantly higher for *EstT* in this model and significantly lower for AST for tilmicosin alone.

Table S7. Sensitivity (Se) and specificity (Sp) estimates for detection of macrolide-resistant *M. haemolytica* from deep nasopharyngeal swab samples collected from feedlot calves. Estimates compare antimicrobial susceptibility testing (AST) results for tulathromycin and tilmicosin resistance with metagenomic sequencing detection of ARGs *msrE-mphE* and *EstT*, using two-test, three-population Bayesian latent class models without informative priors (*n*=909).

Model	Metric	Method	Intermediate as susceptible		Intermediate as resistant	
			**Median**	**95% CrI**	**Median**	**95% CrI**
AST: TULA<br>ARG: *msrE-mphE*	Sensitivity	AST	0.80	0.73, 0.87	0.85	0.78, 0.91
		msrE-mphE	0.62	0.56, 0.69	0.60	0.54, 0.66
	Specificity	AST	0.998	0.99, 0.999	0.998	0.99, 0.999
		msrE-mphE	0.98	0.96, 0.99	0.98	0.96, 0.99
AST: TILM<br>ARG: *EstT*	Sensitivity	AST	0.23	0.10, 0.38	0.52	0.38, 0.67
		EstT	0.43	0.21, 0.65	0.14	0.09, 0.19
	Specificity	AST	0.998	0.99, 0.999	0.997	0.99, 0.999
		EstT	0.99	0.98, 0.999	0.99	0.98, 0.999

CrI – credible interval; AST – antimicrobial susceptibility testing; ARG – antimicrobial resistance gene; seq – long-read metagenomic sequencing; TULA – tulathromycin; TILM – tilmicosin.

Calculation of positive and negative predictive values based on the results of the Bayesian latent class models

Table S8a. Positive and negative predictive values for detection of bacteria from long-read metagenomics estimated based on sensitivity and specificity from Bayesian latent class models.

Inputs:		Inputs:	
Test	***M. haemolytica* 2020**	Test	***M. haemolytica* 2021**
**Sensitivity**	**0.71**	**Sensitivity**	**0.91**
**Specificity**	**0.92**	**Specificity**	**0.90**

	Prior probability of infection	Positive Predictive Value	Negative Predictive Value		Prior probability of infection	Positive Predictive Value	Negative Predictive Value
1	**0**	0.00	1.00	1	**0**	0.00	1.00
2	**0.01**	0.08	1.00	2	**0.01**	0.08	1.00
3	**0.05**	0.32	0.98	3	**0.05**	0.32	0.99
4	**0.1**	0.50	0.97	4	**0.1**	0.50	0.99
5	**0.15**	0.61	0.95	5	**0.15**	0.62	0.98
6	**0.2**	0.69	0.93	6	**0.2**	0.69	0.98
7	**0.25**	**0.75**	0.90	7	**0.25**	**0.75**	0.97
8	**0.3**	0.79	0.88	8	**0.3**	0.80	0.96
9	**0.4**	0.86	0.83	9	**0.4**	0.86	0.94
10	**0.5**	0.90	**0.76**	10	**0.5**	0.90	**0.91**
11	**0.6**	0.93	0.68	11	**0.6**	0.93	0.87
12	**0.7**	0.95	0.58	12	**0.7**	0.96	0.81
13	**0.75**	0.96	0.51	13	**0.75**	0.96	0.77
14	**0.8**	0.97	0.44	14	**0.8**	0.97	0.71
15	**0.85**	0.98	0.36	15	**0.85**	0.98	0.64
16	**0.9**	0.99	0.26	16	**0.9**	0.99	0.53
17	**0.95**	0.99	0.14	17	**0.95**	0.99	0.34
18	**0.99**	1.00	0.03	18	**0.99**	1.00	0.09
19	**1**	1.00	0.00	19	**1**	1.00	0.00

*predictive values > 75% shaded **PPV and NPV values > 75% consistent across both study years were shaded more darkly.
Se and Sp from Table 5.

Table S8b. Positive and negative predictive values for detection of bacteria from long-read metagenomics estimated based on sensitivity and specificity from Bayesian latent class models (continued).

Inputs:		Inputs:	
Test	***P. multocida* 2020**	Test	***P. multocida* 2021**
**Sensitivity**	**0.96**	**Sensitivity**	**0.89**
**Specificity**	**0.98**	**Specificity**	**0.97**

	**Prior probability of infection**	**Positive Predictive Value**	**Negative Predictive Value**		**Prior probability of infection**	**Positive Predictive Value**	**Negative Predictive Value**
1	0	0.00	1.00	1	0	0.00	1.00
2	0.01	0.33	1.00	2	0.01	0.23	1.00
3	0.05	0.72	1.00	3	0.05	0.61	0.99
4	0.1	0.84	1.00	4	0.1	0.77	0.99
5	0.15	0.89	0.99	5	0.15	0.84	0.98
6	0.2	0.92	0.99	6	0.2	0.88	0.97
7	0.25	0.94	0.99	7	0.25	0.91	0.96
8	0.3	0.95	0.98	8	0.3	0.93	0.95
9	0.4	0.97	0.97	9	0.4	0.95	0.93
10	0.5	0.98	0.96	10	0.5	0.97	0.90
11	0.6	0.99	0.94	11	0.6	0.98	0.85
12	0.7	0.99	0.91	12	0.7	0.99	0.79
13	0.75	0.99	0.89	13	0.75	0.99	0.75
14	0.8	0.99	0.86	14	0.8	0.99	0.69
15	0.85	1.00	0.81	15	0.85	0.99	0.61
16	0.9	1.00	0.73	16	0.9	1.00	0.49
17	0.95	1.00	0.56	17	0.95	1.00	0.32
18	0.99	1.00	0.20	18	0.99	1.00	0.08
19	1	1.00	0.00	19	1	1.00	0.00

*predictive values > 75% shaded **PPV and NPV values > 75% consistent across both study years were shaded more darkly.
Se and Sp from Table 5.

Table S8c. Positive and negative predictive values for detection of bacteria from long-read metagenomics estimated based on sensitivity and specificity from Bayesian latent class models (continued).

Inputs:		Inputs:	
Test	***H. somni* 2020**	Test	***H. somni* 2021**
**Sensitivity**	**0.52**	**Sensitivity**	**0.86**
**Specificity**	**0.90**	**Specificity**	**0.97**

	Prior probability of infection	Positive Predictive Value	Negative Predictive Value		Prior probability of infection	Positive Predictive Value	Negative Predictive Value
1	**0**	0.00	1.00	1	**0**	0.00	1.00
2	**0.01**	0.05	0.99	2	**0.01**	0.22	1.00
3	**0.05**	0.21	0.97	3	**0.05**	0.60	0.99
4	**0.1**	0.37	0.94	4	**0.1**	0.76	0.98
5	**0.15**	0.48	0.91	5	**0.15**	0.84	0.98
6	**0.2**	0.57	0.88	6	**0.2**	0.88	0.97
7	**0.25**	0.63	0.85	7	**0.25**	0.91	0.95
8	**0.3**	0.69	**0.81**	8	**0.3**	0.92	**0.94**
9	**0.4**	**0.78**	0.74	9	**0.4**	**0.95**	0.91
10	**0.5**	0.84	0.65	10	**0.5**	0.97	0.87
11	**0.6**	0.89	0.56	11	**0.6**	0.98	0.82
12	**0.7**	0.92	0.45	12	**0.7**	0.99	0.75
13	**0.75**	0.94	0.38	13	**0.75**	0.99	0.70
14	**0.8**	0.95	0.32	14	**0.8**	0.99	0.63
15	**0.85**	0.97	0.25	15	**0.85**	0.99	0.55
16	**0.9**	0.98	0.17	16	**0.9**	1.00	0.44
17	**0.95**	0.99	0.09	17	**0.95**	1.00	0.27
18	**0.99**	1.00	0.02	18	**0.99**	1.00	0.07
19	**1**	1.00	0.00	19	**1**	1.00	0.00

*predictive values > 75% shaded **PPV and NPV values > 75% consistent across both study years were shaded more darkly.
Se and Sp from Table 5.

Table S9a. Positive and negative predictive values for detection of ARGs associated with respiratory bacteria by long-read metagenomics based on sensitivity and specificity from Bayesian latent class models.

Inputs:		Inputs:	
Test	**Any macrolide: *msrE-mphE* 2020**	Test	**Any macrolide: *msrE-mphE* 2021**
**Sensitivity**	**0.61**	**Sensitivity**	**NA**
**Specificity**	**0.97**	**Specificity**	**NA**

	**Prior probability of infection**	**Positive Predictive Value**	**Negative Predictive Value**		**Prior probability of infection**	**Positive Predictive Value**	**Negative Predictive Value**
1	**0**	0.00	1.00	1	**0**	-	-
2	**0.01**	0.17	1.00	2	**0.01**	-	-
3	**0.05**	0.52	0.98	3	**0.05**	-	-
4	**0.1**	0.69	0.96	4	**0.1**	-	-
5	**0.15**	**0.78**	0.93	5	**0.15**	-	-
6	**0.2**	0.84	0.91	6	**0.2**	-	-
7	**0.25**	0.87	0.88	7	**0.25**	-	-
8	**0.3**	0.90	0.85	8	**0.3**	-	-
9	**0.4**	0.93	**0.79**	9	**0.4**	-	-
10	**0.5**	0.95	0.71	10	**0.5**	-	-
11	**0.6**	0.97	0.62	11	**0.6**	-	-
12	**0.7**	0.98	0.52	12	**0.7**	-	-
13	**0.75**	0.98	0.45	13	**0.75**	-	-
14	**0.8**	0.99	0.38	14	**0.8**	-	-
15	**0.85**	0.99	0.30	15	**0.85**	-	-
16	**0.9**	0.99	0.22	16	**0.9**	-	-
17	**0.95**	1.00	0.12	17	**0.95**	-	-
18	**0.99**	1.00	0.02	18	**0.99**	-	-
19	**1**	1.00	0.00	19	**1**	-	-

*predictive values > 75% shaded **PPV and NPV values > 75% consistent across both study years were shaded more darkly.
Se and Sp from Table 7.

Table S9b. Positive and negative predictive values for detection of ARGs associated with respiratory bacteria by long-read metagenomics based on sensitivity and specificity from Bayesian latent class models (continued).

Inputs:		Inputs:	
Test	**Any macrolide: *EstT* 2020**	Test	**Any macrolide: *EstT* 2021**
**Sensitivity**	**0.13**	**Sensitivity**	**0.86**
**Specificity**	**0.98**	**Specificity**	**0.98**

	Prior probability of infection	Positive Predictive Value	Negative Predictive Value		Prior probability of infection	Positive Predictive Value	Negative Predictive Value
1	**0**	0.00	1.00	1	**0**	0.00	1.00
2	**0.01**	0.06	0.99	2	**0.01**	0.46	1.00
3	**0.05**	0.25	0.96	3	**0.05**	0.82	0.99
4	**0.1**	0.42	0.91	4	**0.1**	0.91	0.98
5	**0.15**	0.53	0.86	5	**0.15**	0.94	0.98
6	**0.2**	0.62	0.82	6	**0.2**	0.96	0.97
7	**0.25**	0.68	**0.77**	7	**0.25**	0.97	**0.96**
8	**0.3**	0.74	0.72	8	**0.3**	0.97	0.94
9	**0.4**	**0.81**	0.63	9	**0.4**	**0.98**	0.91
10	**0.5**	0.87	0.53	10	**0.5**	0.99	0.88
11	**0.6**	0.91	0.43	11	**0.6**	0.99	0.82
12	**0.7**	0.94	0.33	12	**0.7**	1.00	0.75
13	**0.75**	0.95	0.27	13	**0.75**	1.00	0.70
14	**0.8**	0.96	0.22	14	**0.8**	1.00	0.64
15	**0.85**	0.97	0.17	15	**0.85**	1.00	0.56
16	**0.9**	0.98	0.11	16	**0.9**	1.00	0.44
17	**0.95**	0.99	0.06	17	**0.95**	1.00	0.27
18	**0.99**	1.00	0.01	18	**0.99**	1.00	0.07
19	**1**	1.00	0.00	19	**1**	1.00	0.00

*predictive values > 75% shaded **PPV and NPV values > 75% consistent across both study years were shaded more darkly.
Se and Sp from Table 7.

Table S9c. Positive and negative predictive values for detection of ARGs associated with respiratory bacteria by long-read metagenomics based on sensitivity and specificity from Bayesian latent class models (continued).

Inputs:			
Test	**Tetracyclines: *tet(H)* 2020**	Test	**Tetracyclines: *tet(H)* 2021**
**Sensitivity**	**0.85**	**Sensitivity**	**0.74**
**Specificity**	**0.91**	**Specificity**	**0.99**

	**Prior probability of infection**	**Positive Predictive Value**	**Negative Predictive Value**		**Prior probability of infection**	**Positive Predictive Value**	**Negative Predictive Value**
1	**0**	0.00	1.00	1	**0**	0.00	1.00
2	**0.01**	0.09	1.00	2	**0.01**	0.43	1.00
3	**0.05**	0.33	0.99	3	**0.05**	0.80	0.99
4	**0.1**	0.51	0.98	4	**0.1**	0.89	0.97
5	**0.15**	0.62	0.97	5	**0.15**	0.93	0.96
6	**0.2**	0.70	0.96	6	**0.2**	0.95	0.94
7	**0.25**	**0.76**	0.95	7	**0.25**	**0.96**	0.92
8	**0.3**	0.80	0.93	8	**0.3**	0.97	0.90
9	**0.4**	0.86	0.90	9	**0.4**	0.98	0.85
10	**0.5**	0.90	**0.86**	10	**0.5**	0.99	**0.79**
11	**0.6**	0.93	0.80	11	**0.6**	0.99	0.72
12	**0.7**	0.96	0.72	12	**0.7**	0.99	0.62
13	**0.75**	0.97	0.67	13	**0.75**	1.00	0.56
14	**0.8**	0.97	0.60	14	**0.8**	1.00	0.49
15	**0.85**	0.98	0.52	15	**0.85**	1.00	0.40
16	**0.9**	0.99	0.40	16	**0.9**	1.00	0.30
17	**0.95**	0.99	0.24	17	**0.95**	1.00	0.17
18	**0.99**	1.00	0.06	18	**0.99**	1.00	0.04
19	**1**	1.00	0.00	19	**1**	1.00	0.00

* predictive values > 75% shaded   **PPV and NPV values > 75% consistent across both study years were shaded more darkly.
Se and Sp from Table 7.

1. Clinical and Laboratory Standards Institute. *Performance standards for antimicrobial disk and dilution susceptibility tests for bacterial isolated from animals.*, 6th ed. CLSI Supplement VET01S ed.; The Clinical and Laboratory Standards Institute: Wayne, PA, USA, 2023.
2. Dhindwal, P.; Thompson, C.; Kos, D.; Planedin, K.; Jain, R.; Jelinski, M.; Ruzzini, A. A neglected and emerging antimicrobial resistance gene encodes for a serine-dependent macrolide esterase. *Proc Natl Acad Sci U S A* **2023**, *120*, e2219827120, doi:10.1073/pnas.2219827120.
3. Desmolaize, B.; Rose, S.; Wilhelm, C.; Warrass, R.; Douthwaite, S. Combinations of macrolide resistance determinants in field isolates of Mannheimia haemolytica and Pasteurella multocida. *Antimicrob Agents Chemother* **2011**, *55*, 4128-4133, doi:10.1128/aac.00450-11.
